# A Molecularly Detailed Na_V_1.5 Model Reveals a New Class I Antiarrhythmic Target

**DOI:** 10.1016/j.jacbts.2019.06.002

**Published:** 2019-10-28

**Authors:** Jonathan D. Moreno, Wandi Zhu, Kathryn Mangold, Woenho Chung, Jonathan R. Silva

**Affiliations:** a Division of Cardiology, Department of Medicine, Washington University in St. Louis, St. Louis, Missouri; b Department of Biomedical Engineering, Washington University in St. Louis, St. Louis, Missouri

**Keywords:** arrhythmias, computational biology, ion channels, pharmacology, translational studies, APD, action potential duration, BCL2000, basic cycle length of 2,000 ms, DIII-VSD, domain III voltage-sensing domain, EAD, early afterdepolarization, IC_50_, half-maximal inhibitory voltage, LQT3, long QT syndrome type 3, RFI, recovery from inactivation, SSA, steady-state availability, UDB, use-dependent block, V_1/2_, half-maximal voltage, VSD, voltage-sensing domain

## Abstract

Antiarrhythmic treatment strategies remain suboptimal due to our inability to predict how drug interactions with ion channels will affect the ability of the tissues to initiate and sustain an arrhythmia. We built a multiscale molecular model of the Na^+^ channel domain III (domain III voltage-sensing domain) to highlight the molecular underpinnings responsible for mexiletine drug efficacy. This model predicts that a hyperpolarizing shift in the domain III voltage-sensing domain is critical for drug efficacy and may be leveraged to design more potent Class I molecules. The model was therefore used to design, in silico, a theoretical mexiletine booster that can dramatically rescue a mutant resistant to the potent antiarrhythmic effects of mexiletine. Our framework provides a strategy for in silico design of precision-targeted therapeutic agents that simultaneously assesses antiarrhythmic markers of success and failure at multiple spatial and time scales. This approach provides a roadmap for the design of novel molecular-based therapy to treat myriad arrhythmia syndromes, including ventricular tachycardia, heart failure arrhythmias, and inherited arrhythmia syndromes.

## Highlights

• Antiarrhythmic therapies remain suboptimal due to our inability to predict how drug interactions with ion channels will affect the ability of the tissue to initiate and sustain an arrhythmia.
• We built a computational framework that allows for in silico design of precision-targeted therapeutic agents that simultaneously assesses antiarrhythmic markers of success and failure at multiple spatial and time scales. Using this framework, a novel in silico mexiletine “booster” was designed that may dramatically improve the efficacy of mexiletine in suppression of arrhythmia triggers.
• These results provide a roadmap for the design of novel molecular-based therapy to treat myriad arrhythmia syndromes, including ventricular tachycardia, heart failure arrhythmias, and inherited arrhythmia syndromes.
• In summary, computational modeling approaches to drug discovery represent a novel tool to design and test precision-targeted therapeutic agents. By exploiting nontraditional ion channel drug targets, an entirely new dimension can be added to the wide parameter space of traditional antiarrhythmic drugs to develop more precision-targeted and potent Class I therapeutic agents.

## Visual Abstract

**Figure:**
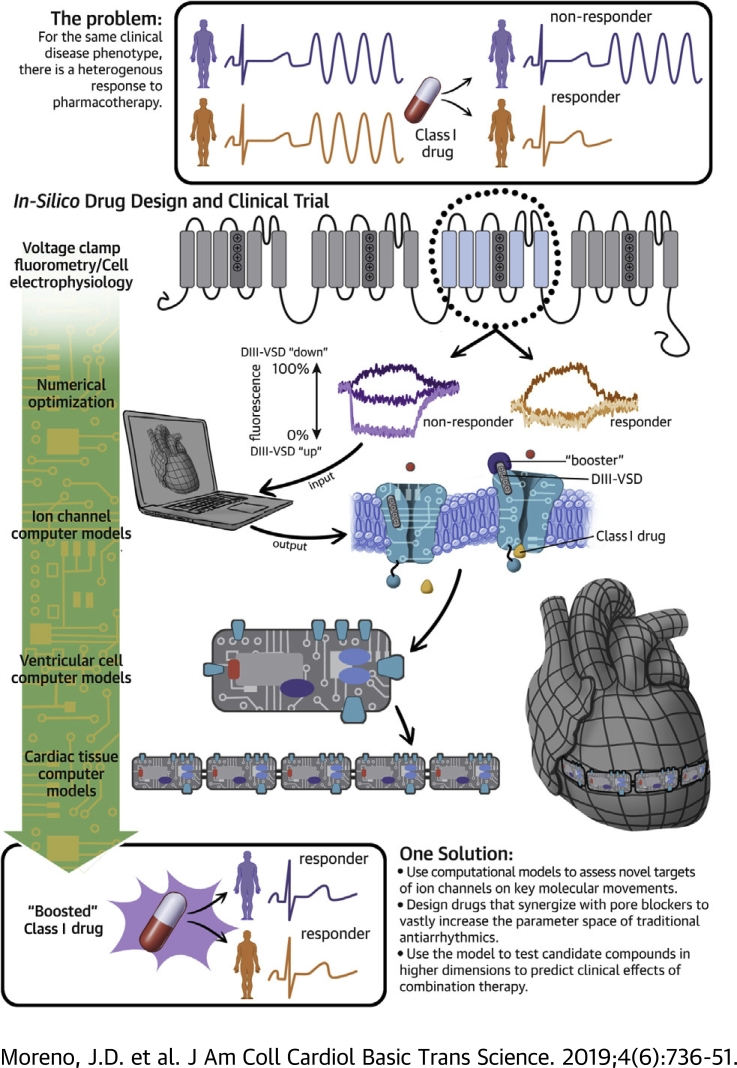

Current antiarrhythmic treatment strategies remain suboptimal due to our inability to predict patient-specific responses. As evidenced by large clinical trials (e.g., CAST [Cardiac Arrhythmia Suppression Trial] [1], SWORD [Survival With Oral d-Sotalol] [2]), antiarrhythmic therapy can paradoxically increase arrhythmia burden compared with placebo and lead to increased risk of death. This outcome is due, in part, to the complex kinetics of the drug channel interaction that includes strong bidirectional feedback between how drugs alter the action potential waveform affecting voltage-dependent potency, as well as electrotonic coupling in tissue, which we (3) and others (4) have shown can lead to an even more complex response to drugs that may not be appreciated in single-cell studies. For both acquired and inherited arrhythmia syndromes, this scenario leads to a dangerous trial-and-error approach to choosing appropriate pharmacotherapy for patients. Furthermore, despite an enormous amount of research into antiarrhythmic drug therapy that has produced molecules with a wide range of pharmacokinetic properties, they all block a single target: the channel pore. Thus, targets of ion channels other than the channel pore may add an entirely new dimension to antiarrhythmic drug therapy.

Structurally, the alpha-subunit of Na^+^ channels is formed by a monomer with 4 homologous domains (DI to DIV), each with 6 transmembrane subunits (S1 to S6). The S1-S4 of each domain forms the voltage-sensing domain (VSD), and S5-S6 forms the pore 5, 6. Upon membrane depolarization, the VSDs activate and open the pore, allowing Na^+^ entry into the cell. Experimental recordings that monitor VSD conformation changes (voltage clamp fluorometry) have shown that VSD movement is modulated by binding of local anesthetics, and that when lidocaine binds to the Na^+^ channel, it stabilizes the domain III VSD (DIII-VSD) in an activated conformation (7). Our recent experimental results (8) show that DIII-VSD dynamics significantly regulate mexiletine blockade of Na_V_1.5, and the differential response of long QT syndrome type 3 (LQT3) carriers to mexiletine is due, in large part, to mutation-specific VSD dynamics.

To date, the myriad parameters within Na^+^ channel kinetic models have been shown to have a significant impact on the ability of the heart to initiate and sustain an arrhythmia, and exploiting these parameters has been useful for understanding Na^+^ channel pharmacology (9). However, it has not yet been possible to design targeted interventions that alter these parameters because they are not specifically connected to the channel structure.

The current study used our experimental results (8) to develop a computational model that tracks molecular DIII-VSD movement, Na^+^ channel electrophysiology, and the response of both to mexiletine drug blockade for 2 LQT3 mutations: R1626P, shown to be mexiletine sensitive, and M1652R, shown to be mexiletine resistant. The fidelity of the model for these 2 mutants provides confidence that the relation between the DIII-VSD and channel gating is well represented by the model and allows us to predict novel therapeutic approaches based on this relation. This finding is clinically important; despite the common use of mexiletine in treating ischemia-related ventricular tachycardia in those without Na^+^ channel mutations (WT), its effectiveness is suboptimal, necessitating high clinical doses, and plagued by numerous side effects. Our previous study confirms this lack of efficacy in WT channels.

To our knowledge, this is the first such multiscale computational model that explicitly displays the experimentally parameterized molecular underpinnings of drug efficacy from channel kinetics to higher dimensional cardiac fibers 10, 11. Using the model as a therapeutic prediction tool, we then developed an in silico mexiletine “booster”: a theoretical drug that alters DIII-VSD activation kinetics and significantly enhances drug efficacy. We propose that combination therapy with common pore blockers, enhanced by allosteric channel modulation, could dramatically alter the landscape for antiarrhythmic therapy by adding another dimension to the parameter space of drug efficacy. This approach would expand the number of patients who would receive clinical benefit from existing therapeutic agents and allow for a lower concentration of drugs to be used, decreasing off-target side effects.

## Methods

Computational Markov models of the WT, M1652R, and R1626P LQT3 mutants were formulated with and without the mexiletine drug channel interaction via numerical optimization from experimental data, as previously described 12, 13. These models include both channel kinetics and voltage-clamp fluorescence describing the DIII-VSD movement. The drug channel model was incorporated into a computational model of the human ventricular myocyte (14) to assess cellular and tissue response to drug therapy. Our model incorporates experimental data from HEK cells and from the *Xenopus* cell expression system where appropriate.

Results for experimental data are expressed as mean ± SEM. Significance between groups was tested by using the Student’s *t*-test. We have used a variety of figure types to best display the experimental data. The model-fitting plots are scatter plots of the summary experimental data (points, experiment) with the simulated model fits shown as linear overlays. The tissue data are displays of cellular action potential as well as simulated individual currents (e.g., Na^+^ current). Summary data for the biotin experiments are shown as bar plots. Detailed methods are available in the Supplemental Information.

## Results

### Biophysical characteristics of 2 LQT3 mutants

We focused on the R1626P (RP) and the M1652R (MR) mutations because of their marked differential responses to mexiletine. Both mutations lie within the DIV S4 segment and produce an increased late Na^+^ current ∼0.7% to 1.0% of the peak current. Aside from a ∼15 mV depolarizing shift in steady-state availability (SSA) for the MR mutation, and a ∼8 mV hyperpolarization of the RP mutation, the electrophysiology of both mutations is similar (15). At resting membrane potential, however, the DIII-VSD of RP is nearly ∼90% activated (“up” position), whereas the MR DIII-VSD is only ∼50% activated (8).

### Drug-free model development

We began by developing drug-free models of both mutations as well as wild type, which were easily fit with a well-established 8-state Markov model (3). The late currents of the mutations were simulated as slowly inactivating currents and were numerically optimized to yield 0.1%, 0.67%, and 1% late current (ratio to the peak current) for the WT, M1652R, and R1626P constructs, respectively (15). We then focused on expanding the kinetic model to account for DIII-VSD movement, shown to be stabilized in an active conformation by mexiletine (8). Using voltage-clamp fluorometry methods previously described (16), the kinetics of DIII-VSD were simulated in response to channel activation. Briefly, by attaching a fluorescent tag to the DIII-VSD and expressing Na_V_1.5 in a *Xenopus* oocyte cell expression system, we could simultaneously record current kinetics and DIII-VSD movement 16, 17.

**Figure 1:**
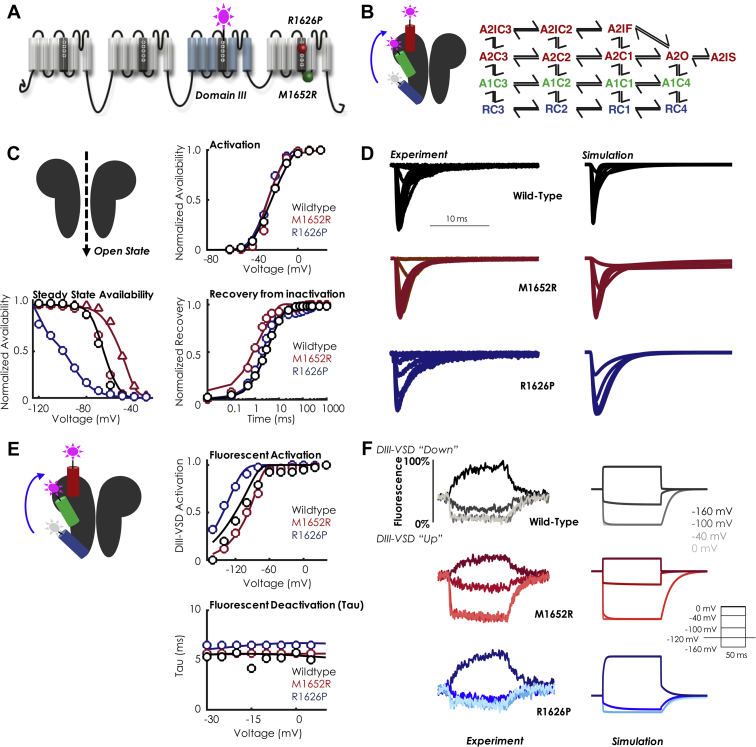
Model Schematic With Drug-Free Kinetics **(A)** Topology of the Na_V_1.5 channel and location of the 2 long QT mutations with distinct mexiletine sensitivity, R1626P (**red ball**, sensitive) and M1652R (**green ball**, insensitive). A fluorophore is attached to domain III (DIII). **(B)** Schematic representation of the DIII voltage-sensing domain (DIII-VSD) in the Na^+^ channel and corresponding Markov state-chain diagram of the drug-free Na^+^ channel. The **blue** DIII-VSD is in a “down” or rested position and corresponds to the **blue** “R” states in the Markov model **(far right)**. The **green** DIII-VSD represents the first transition to a fluorescent regime labeled “A1” (first activated) and corresponds to the **green** “A1” states in the Markov model. The second transition to A2 is denoted with the **red** DIII-VSD, which represents a “fully up” state and corresponds to the “A2” states. As depicted, the model includes 16 states: 4 rested states (“R”), 4 first-activated states (“A1”), and 8 second-activated states (“A2”). The rate constants governing the transitions can be found in the Supplemental Material. **(C)** Current kinetics. In each panel, the **points** represent the experimental data, and the **solid lines** represent the model fits to the data. WT is shown in **black**, M1652R is shown in **red**, and R1626P is shown in **blue**. The **triangles** in the steady-state availability (SSA) plot indicate the HEK transformation of the SSA, detailed in the Supplemental Material. The model fits to current kinetics include steady-state availability, activation, recovery from inactivation, Tau of deactivation (not shown), and mean open time (for WT). Details of the protocols are given in the Supplemental Material. **(D)** Representative traces of currents in the 3 constructs. Shown at **left** is the experiment, **right** is the simulation. The channel was held at –120 mV to steady state and then pulsed to 0 mV for 25 ms. WT is in **black**, M1652R is in **red**, and R1626P is in **blue**. **(E)** Fluorescent kinetics. In each panel, the **points** represent the experimental data, and the **solid lines** represent the model fits to the data. WT is shown in **black**, M1652R is shown in **red**, and R1626P is shown in **blue**. Shown are fluorescent activation and deactivation. **(F)** Representative traces of fluorescence in the 3 constructs (WT, **black**; M1652R, **red**; R1626P, **blue**). Shown at **left** is the experiment, **right** is the simulation for 4 different test voltages (–160 mV, –100 mV, –40 mV, and 0 mV; the voltage protocol is shown in the **inset**). The experimental data for steady-state activation and recovery from inactivation are from Ruan et al. (15); the experimental data for SSA, fluorescent activation, and fluorescent tau are from Zhu et al. (8).

As can be seen in Figure 1E, much of the DIII-VSD movement occurs in a voltage range before Na^+^ channels begin to activate. At –80 mV, the RP DIII-VSD is ∼90% in the active position, whereas the MR DIII-VSD is only ∼50% activated. Experimental data showing multiple time constants in response to depolarizing pulses of different durations suggest at least 2 active conformations of the DIII-VSD 16, 18. Therefore, to best fit the fluorescent data, 3 regimes were added to the model: a row of resting closed states (RC1 – RC4) which represent DIII-VSD in a “down” position; a first-activated regime (A1C3 – A1C4), which represents the first activation of the DIII-VSD (DIII “moving up”); and a second-activated regime (A2 states) which represents the second-activation step of DIII-VSD (DIII “fully up”) and contains the top 8 states. The full 16-state drug-free model is shown in Figure 1B. Fluorescence was then simulated by plotting the ratio of the sum of the A1 and A2 states to the total states. As detailed in the Supplemental Material, analysis of the half-maximal voltage (V_1/2_) of DIII-VSD activation versus tonic block with mexiletine allowed us to tease out the relative fluorescent contributions of the A1 and A2 states (Supplemental Figure 7).

One benefit of using a computational approach is that data from different expression systems can be reconciled by altering the appropriate parameters. For example, in simulating MR SSA, we chose to incorporate a 15 mV depolarizing shift of MR compared with WT to more closely simulate the results obtained by us and others (15) in the HEK expression system. Thus, in Figure 1C (SSA), the red circles represent data obtained in *Xenopus* oocytes, the red triangles represent the “HEK-transformed” SSA curve (+15 mV depolarization), and the solid red line represents the model fit. Overall, Figure 1 shows that the resultant simulations (solid lines) match closely with the experimental data (points) and capture a wide range of channel kinetics.

Figures 1D and 1F present a side-by-side comparison of the simulated Na^+^ channel currents and fluorescence traces in response to a voltage step protocol. As can be seen, for both mutations and WT, the activation of DIII-VSD to depolarized potentials is rapid and on the order of 3 to 5 ms, similar to current activation. At a membrane potential of –120 mV, WT is ∼50% activated (“up”), MR is ∼25% activated (“up”), and RP is ∼75% activated (“up”). In general, over the physiological voltage range, the RP mutation traps the DIII-VSD in a relatively activated position, a necessary prerequisite for drug binding (discussed in the following section).

Experimentally, maximal fluorescence occurs at the most hyperpolarized potentials, which implies that the fluorescent molecule, TAMRA-MTS, is being quenched at elevated membrane voltages. Thus, DIII-VSD activation is inversely proportional to fluorescence, and the plots are normalized to the range of minimal to maximal fluorescence. As such, Figure 1E is labeled as “DIII-VSD activation” (which is equivalent to the DIII-VSD position in the membrane). This is congruent with Figure 1F labeling: at –160 mV, all constructs fluoresce maximally, shown as the upward deflection of the fluorescence curve. Upon depolarization, the fluorescence is quenched to 0%, as DIII-VSD is in an “up” position.

### Mexiletine drug-bound model development

We next expanded the model to account for mexiletine drug binding. Our previous results suggested that the voltage dependence of DIII-VSD activation strongly correlates with tonic block by mexiletine (Figure 4 of Zhu et al. [8]). Briefly, tonic block, a measure of first-pulse block, is assessed at holding potentials before much closed-state inactivation occurs; thus, the apparent differences in tonic block seen for 15 different mutants were primarily accounted for by the difference in the DIII-VSD. For the RP mutation, the V_1/2_ of DIII-VSD activation is –143 mV and has a 10-fold lower half-maximal inhibitory concentration (IC_50_) for tonic block (69.5 μM), compared with MR, which has a V_1/2_ of –100 mV and a tonic block IC_50_ of 624 μM. Our results, as well as others (15), suggest that the intrinsic affinity of mexiletine to the local anesthetic receptor for the mutants is likely the same, but the measured tonic block differences represent both contamination by the inactivated state and differences in DIII-VSD guarding the receptor. Taken together, we simulated mexiletine drug block as transitions only from the A2 states to the drug-bound states; in other words, DIII-VSD needs to be in a fully activated (A2 regime) position to allow for drug binding.

**Figure 2:**
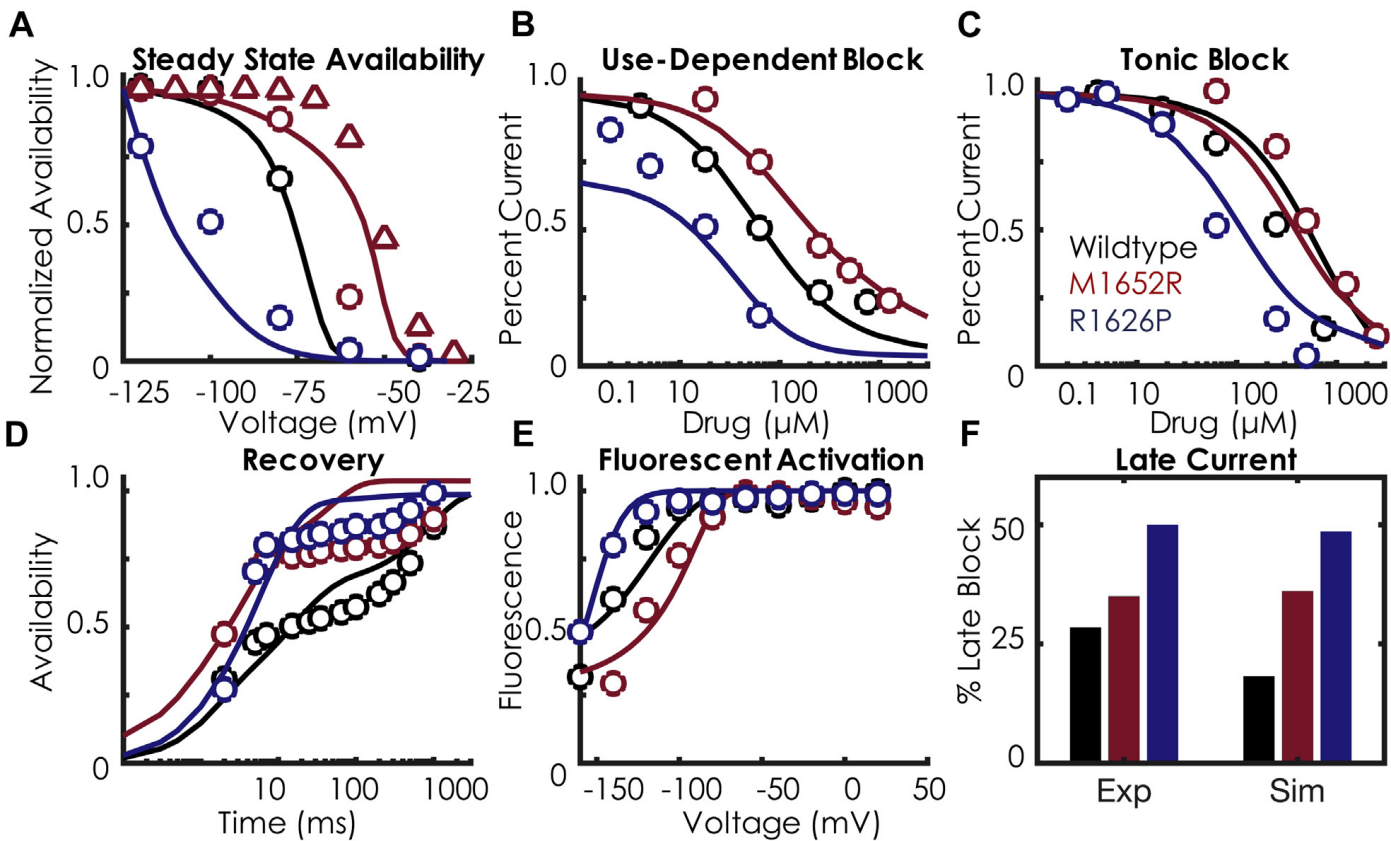
Mexiletine Drug-Binding Kinetics In each panel, the **points** represent the experimental data, and the **solid lines** represent the model fits to the data. WT is shown in **black**, M1652R is shown in **red**, and R1626P is shown in **blue**. **(A)** SSA with mexiletine 250 μM. As in Figure 1, the triangles represent the HEK transformation (Supplemental Material). There is a minimal shift in SSA with mexiletine, indicating minimal inactivated state binding. **(B)** Use-dependent block with mexiletine 0.1 to 1,000 μM. There is a 10 times differential sensitivity to mexiletine between MR **(blue)** and RP **(red)**. **(C)** Tonic block with mexiletine 0.1 to 1,000 μM. The marked differential sensitivity persists for tonic block, similar to the use-dependent block seen in **panel B**. **(D)** Recovery from inactivation at –100 mV with mexiletine 250 μM. **(E)** Fluorescent activation with 4,000 μM mexiletine. Note the marked hyperpolarization of all 3 constructs with mexiletine, indicating that mexiletine stabilizes DIII-VSD in the activated conformation. **(F)** Late block with mexiletine 75 μM. Similar to the experiment, there is increasing affinity of late current block (measured after 400 ms of a depolarizing pulse) between the 3 constructs: WT < M1652R < R1626P. Details of the protocols can be found in the Supplemental Material. Abbreviations as in Figure 1.

The resultant models after numerical optimization (Supplemental Material) are shown in Figure 2, with additional biophysical characterization shown in Supplemental Figure 2. As can be seen, the model simulations fit the experimental data over a wide range of pacing protocols and drug concentrations. Similar to the experimental data, there are marked differences in the affinities of mexiletine between the 2 mutants for both use-dependent block (UDB) and tonic block, with the RP mutation exhibiting a 10 to 15 times increased sensitivity to mexiletine compared with MR (tonic block: 69.5 μM vs. 625 μM; UDB: 16 μM vs. 240 μM). In the model, these differences are largely regulated by the difference in DIII-VSD activation (Figure 2E), given that the intrinsic affinity of mexiletine to the receptor for the 3 models was optimized to a constant affinity (156 μM). Recovery kinetics with mexiletine are similar between the 2 mutants (Figure 2D), which are both faster than WT. Application of mexiletine 4,000 μM stabilizes the DIII-VSD in the activated position (shifts the DIII-VSD to more hyperpolarized potentials) by 13 mV for RP (–156.8 mV vs. –143 mV) and by 22 mV for MR (–122.2 mV vs. –100 mV). Mexiletine application also slows recovery of DIII-VSD fluorescence (Tau_Recovery_: RP 19.05 ms [experiment] vs. 19.02 ms [simulation]; MR 14.2 ms [experiment] vs. 14.2 ms [simulation after a depolarizing pulse to 20 mV). Late current block, the amount of current blocked by mexiletine 75 μM after a 400 ms depolarizing pulse, is shown in Figure 2F. The RP mutation is slightly more sensitive to late current block than MR (50% vs. 35% block).

### Cellular simulations recapitulate differential sensitivities to mexiletine

**Figure 3:**
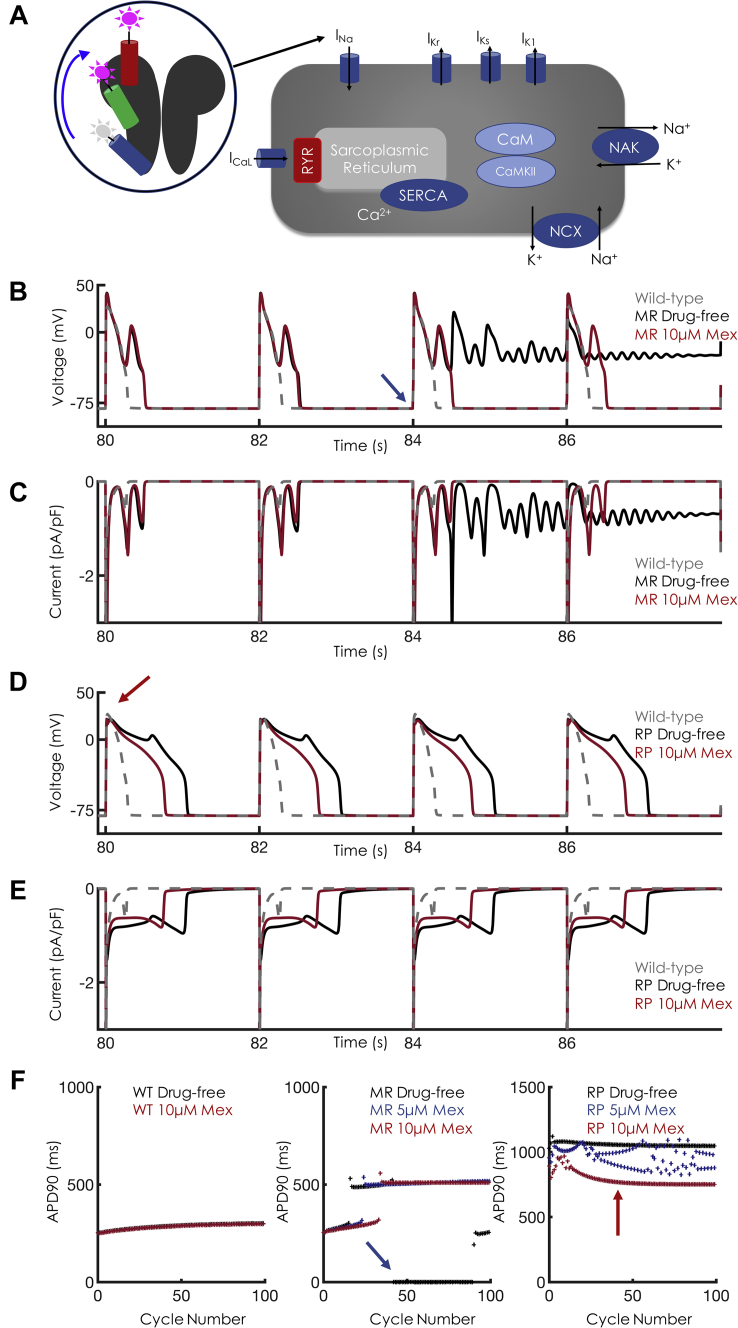
Cellular Level Effects of Mex at Bradycardic Pacing **(A)** Schematic of single-cell simulations. The Markov model representation of the Na^+^ channel was placed into an action potential (AP) model, replacing the standard Hodgkin-Huxley formulation. **(B)** Single-cell APs at basic cycle length 2,000 ms (BCL2000) for M1652R. In the absence of mexiletine (Mex), the M1652R mutation displays chaotic behavior with failure to repolarize **(black trace)**. Application of Mex 10 μM **(red trace)** induced single early afterdepolarizations (EADs) but enhanced repolarization. Wild type, drug free is shown in **gray**. **(C)** Na^+^ currents for the corresponding APs shown in panel B. Note that the drug-free condition induces sustained inward current **(black trace)**. Despite high clinical Mex (10 μM), Na^+^ channels reactivate, which causes the EADs shown in **B**. **(D)** Single-cell APs at BCL2000 for R1626P. In the absence of Mex, the R1626P shows characteristic EADs and a markedly prolonged AP duration (APD, **black trace**). Application of Mex 10 μM **(red)** successfully abolished the EADs, leading to monotonic repolarization but still with a prolonged APD compared with wild type **(gray trace)**. **(E)** Na^+^ currents for the corresponding APs shown in **D**. Note the shortened late inward Na^+^ current with application of Mex. **(F)** APD as a function of cycle number for 100 beats at BCL2000 (corresponding to the APs shown in **B and D**). Note in the M1652R panel, the **blue arrow** at beat 42 corresponds to the **blue arrow in B** depicting the onset of a failure of the repolarization regime. For the R1626P mutation, the **red arrow** corresponds to beat 40, shown in **D**. Note the beat-to-beat APD variability with Mex 5 μM **(blue trace)**. After early chaos (first 10 beats), Mex 10 μM induces monotonic repolarization (absence of EADs), shown as the **red curve in F (far right)**.

The next step was to incorporate our Na^+^ channel models into the Grandi-Bers computational model of the human ventricular myocyte (14), with substitution of our Na^+^ channel model for the baseline formulation and modification of the maximum chloride conductance of the baseline model for more accurate repolarization (Supplemental Material). We focused on a slow-pacing regime of 0.5 Hz (basic cycle length of 2,000 ms [BCL2000]) for our cellular simulations. In the drug-free conditions, both MR and RP display marked prolongation in action potential duration at 90% repolarization (APD_90_), as well as sustained late Na^+^ current (Figures 3B and 3C). Interestingly, the MR mutation displays chaotic behavior, including progressive APD prolongation until the onset of early afterdepolarizations (EADs), a hallmark bradycardic arrhythmia trigger, as well as salvos of sustained depolarization (Figure 3B, Supplemental Figure 4). Application of mexiletine 10 μM (high clinical concentration) is unable to normalize APD_90_: ∼509 ms versus ∼523 ms in the drug-free condition. In contrast, the RP mutation shows stable EADs, with an APD_90_ of ∼1,047 ms in the absence of drug. Application of mexiletine 10 μM is able to decrease the APD_90_ of RP by 28% and abolish the EAD triggers, similar to clinical results (Figure 2 in Ruan et al. [15]). As expected, mexiletine had negligible effects on WT APD_90_. Consistent with other Class Ib antiarrhythmic drugs, mexiletine 10 μM has negligible effects on maximum upstroke velocity (Supplemental Figure 3).

### Development of a mexiletine “booster”

Given mexiletine’s safety and widespread use in the clinic, we hypothesized that combination therapy with mexiletine and a “booster” drug might synergize for more potent antiarrhythmic effects. Because mexiletine sensitivity between RP and MR seems to be driven by the relative position of the DIII-VSD, we hypothesized that by holding the DIII-VSD in an “up” and activated position, we could enhance the efficacy of mexiletine for patients found to be mexiletine resistant (e.g., MR mutants). We thus turned to the computational model to design (i.e., in silico) a mexiletine booster and then tested its efficacy in combination with mexiletine.

**Figure 4:**
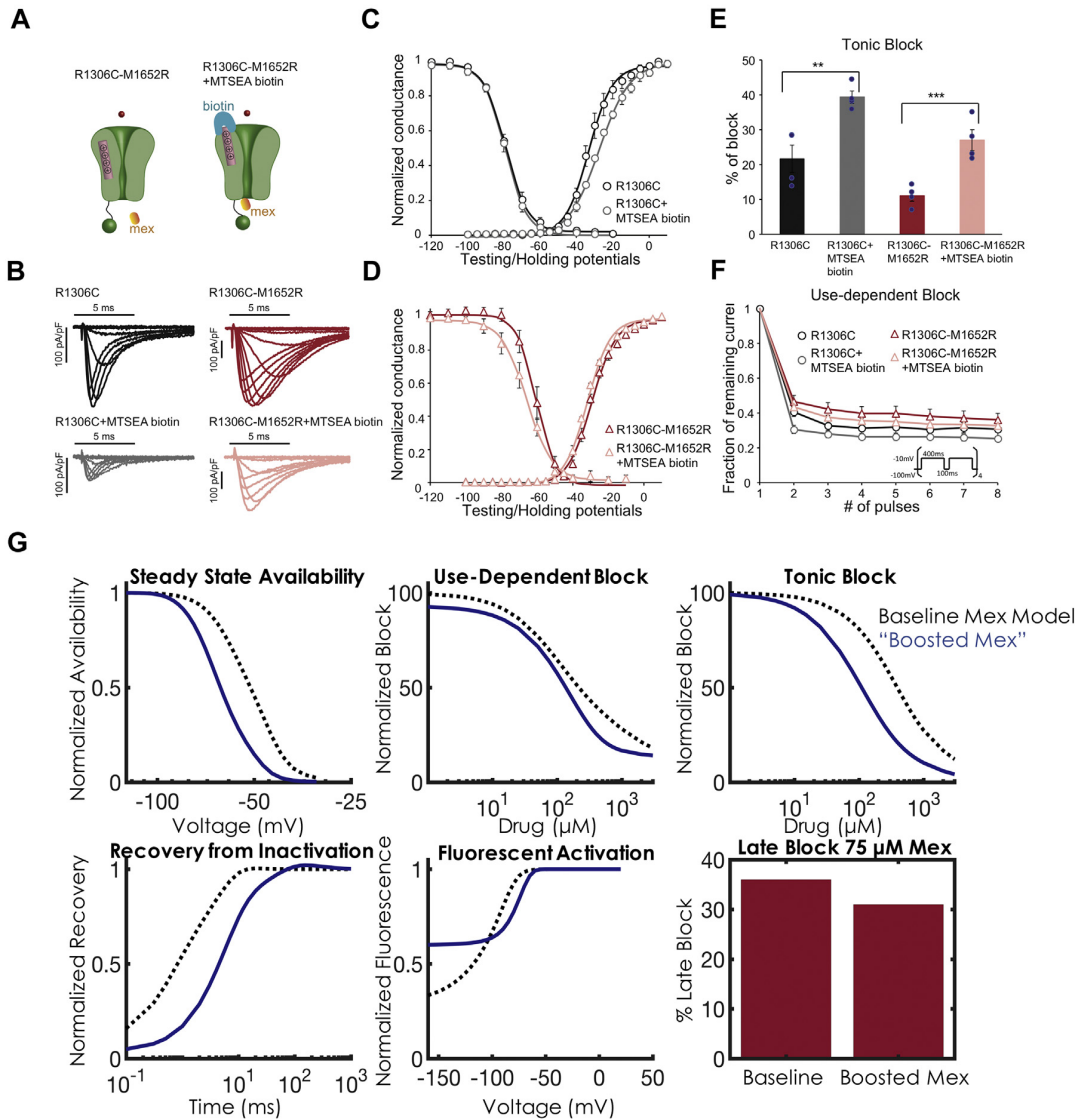
Effects of Biotin on the DIII-VSD and Optimization of a Mex “Booster” **(A)** Schematic of the Na^+^ channel with the double-mutation R1306C M1652R. Application of MTSEA-biotin traps the DIII-VSD **(pink)** in the activated position. **(B)** Representative current traces of the R1306C and R1306C M1652R channels before and 20 min after MTSEA-biotin 20 μM perfusion. Peak current amplitude for both constructs decreased after MTSEA-biotin application. **(C)** Effects of biotin on channel activation (conductance-voltage [GV]) and SSA of the R1306C channel. Application of biotin induces a small right shift in the GV curve. Results are expressed as mean ± SEM from a group of 4 to 6 cells. **(D)** Effect of biotin on GV and SSA of the M1652R R1306C double mutant. Biotin induces a hyperpolarizing shift in SSA, compared with control. **(E)** Summary data of tonic block. Application of MTSEA-biotin to both mutants induces a significant ∼2 times increase in tonic block for Mex 50 μM. Significance was determined by using the Student’s *t*-test. **p < 0.01, ***p < 0.005. **(F)** Use-dependent block (UDB) for Mex 50 μM. UDB is measured by applying eight 400 ms depolarizing pulses at a 2 Hz frequency to mimic a tachycardia condition. Although there was a trend of decreasing availability after biotin for both constructs, the changes were not significant. **(G)** Optimization of a Mex booster. Starting with the optimized parameters from the M1652R mutation (baseline Mex model shown in the **black dashed curve**), the parameters for DIII-VSD movement were optimized to induce a 15 mV hyperpolarization in SSA and a 2-fold increase in tonic block and UDB. After optimization, the Mex booster is shown in **blue**. As in **D**, the “booster” rescues SSA back to WT. UDB **(top middle)** and tonic block **(top right)** exhibit marked increased affinity with the Mex booster **(blue trace)** compared with the baseline Mex model **(black dashed traces)**. Recovery from inactivation **(bottom left)**, fluorescent activation **(bottom middle)**, and late block **(bottom right)** with Mex 75 μM are plotted in the same fashion, but these 3 protocols were not used for optimization. They serve as model predictions (details are given in the text). Abbreviations as in Figures 1 and 3.

This concept was first tested experimentally (Figure 4). We used MTSEA-biotin (biotin) to alter the conformation of the DIII-VSD. It was previously shown that extracellular application of biotin can modulate the cysteine residue at the 1,306 location 7, 19. It stabilizes the DIII-VSD in an activated position in R1306C channels. We engineered the R1306C mutation as a biotin target, as well as an R1306C M1652R double mutation into Na_V_1.5, and expressed the mutant channels in the HEK cell expression system. As shown in these previous studies, application of biotin to R1306C channel decreases peak Na^+^ current, which stabilized ∼20 min after biotin perfusion. The decrease in Na^+^ current amplitude suggests that MTSEA-biotin binds to the cysteine residue and modulates the DIII-VSD conformation. We also observed a reduction in peak Na^+^ current in the double-mutant R1306C M1652R 20 min after biotin application (Figure 4B). Biotin caused a small depolarizing shift in the activation (conductance-voltage [GV]) curve, but no effect on the SSA curve, for the R1306C channel (Figure 4C). Similar to the M1652R channel, the double-mutant R1306C M1652R channel exhibited a large depolarizing shift in the SSA curve compared with the R1306C channel (R1306C V_1/2_ = –79.5 ± 1.2 mV; R1306C M1652R V_1/2_ = –59.0 ± 2.7 mV) (Figures 4C and 4D). This depolarizing shift increases the availability of the channel (gain of function). Notably, when the R1306C M1652R channel was modified with a biotin, the SSA curve was shifted to hyperpolarizing potentials (V_1/2_ = –66.1 ± 3.8 mV). Thus, biotin partially corrects the alteration of the SSA curve caused by the M1652R mutation.

Finally, we tested block by mexiletine 50 μM in R1306C and R1306C M1652R channels modified by biotin. There was a significant 81% and 146% increase in tonic block in the R1306C and R1306C M1652R channels, respectively (Figure 4E). In addition, for the double mutant, mexiletine 50 μM blocks 17.4 ± 6.1% late current; the addition of biotin and mexiletine 50 μM blocks 65.3 ± 5.6% late current (p = 0.004). It is noteworthy that the late current was quantified 50 ms after a depolarization pulse of –40 mV. For mexiletine UDB, there was no significant difference between channels with or without biotin (Figure 4F). This result is possibly due to MTSEA-biotin immobilization of the DIII-VSD in the activated conformation, which eliminates dynamic control of channel gating by the DIII-VSD. Overall, these experiments are congruent with our hypothesis that “pulling up” the DIII-VSD with a small molecule has the potential to enhance mexiletine efficacy.

To design an in silico booster, we modified the rates of the DIII-VSD movement in the drug-free model of MR (i.e., ax, bx, ay, by, a3, b3), while keeping the drug-bound rates constant, to simulate a 15 mV hyperpolarizing shift of SSA (in the absence of mexiletine) and a 2-fold increase in tonic block and UDB in the presence of mexiletine. The results of simulated biotin are shown in Figure 4G. As can be seen, in silico biotin shifts the SSA back to WT and significantly increases both tonic block (IC_50_ 105 μM vs. 400 μM) and UDB (IC_50_ 125 μM vs. 200 μM). We simulated the effects of recovery from inactivation (RFI), DIII fluorescence, and late block as confirmatory validation (e.g., these protocols were not used in the optimization routine). Thus, our model predicts that when DIII-VSD is held up, which shifts SSA, recovery from inactivation is slowed. Furthermore, even at significantly hyperpolarized potentials, DIII-VSD remains in the up and activated position. Interestingly, we found no appreciable increased late current blockade (as a percentage of peak current block), although an examination at the actual Na^+^ current trace (Figure 4G) exhibited markedly less late current compared with drug-free conditions.

**Figure 5:**
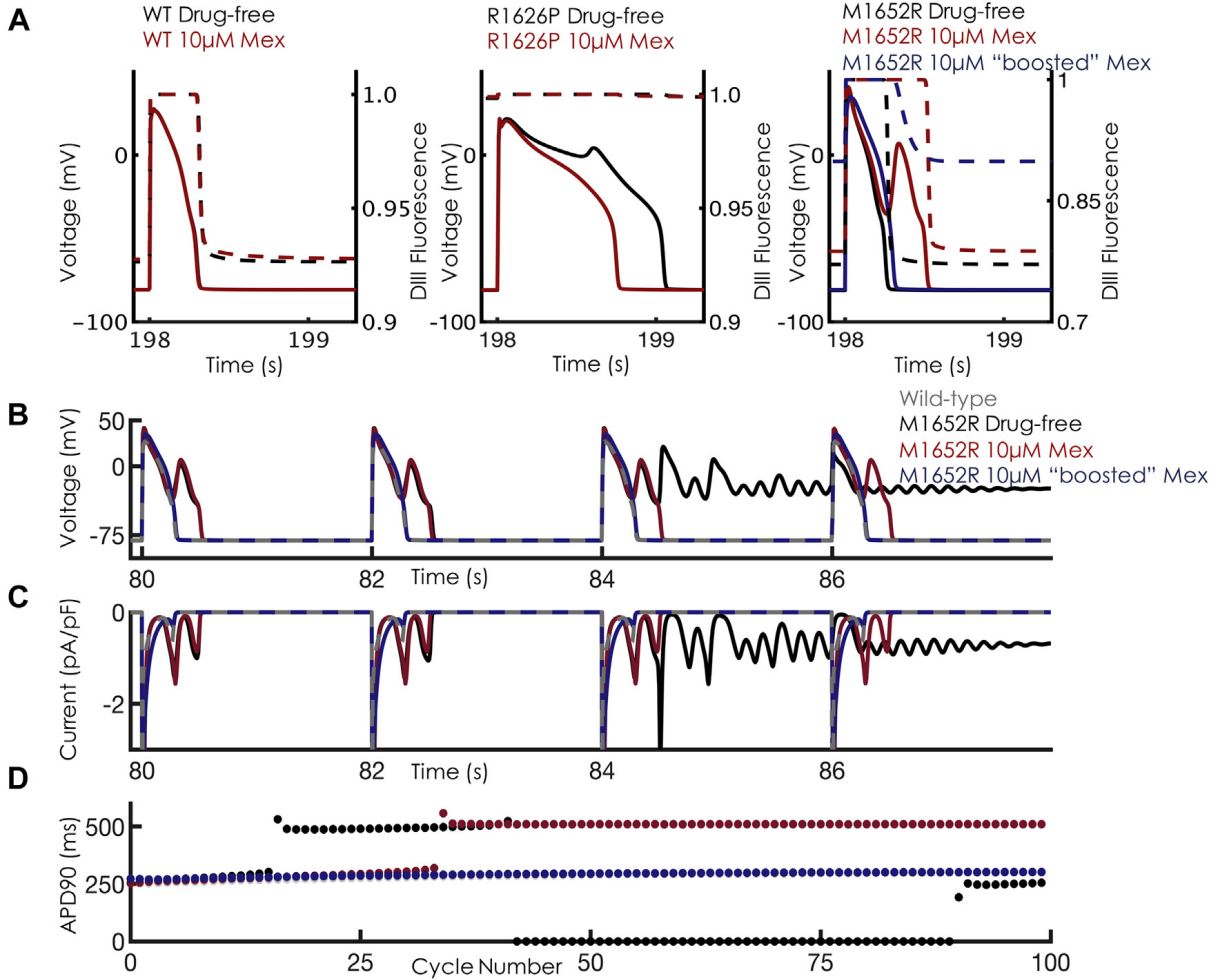
Effects of “Boosted” Mex Cellular Dynamics **(A)** Overlay of APs and DIII-VSD fluorescence in the absence **(black)** and presence **(red)** of Mex 10 μM for WT **(left)**, R1626P **(middle)**, and M1652R **(right)**. In response to voltage depolarization (the AP), the DIII-VSD **(dashed lines)** fluoresces before decaying to its resting value at –80 mV (∼93% for WT). For R1626P, the DIII-VSD remains 100% activated throughout the duration of the AP. In M1652 **(far right)**, the DIII-VSD relaxes to ∼75%, activated both in the presence and absence of Mex. Application of “boosted” Mex “holds up” the DIII-VSD to ∼90% **(dashed blue trace)**. **(B)** The effects of “boosted” Mex (Mex 10 μM + “booster”) on APs. “Boosted” Mex **(shown in blue)** abolishes EAD triggers, failed repolarization, and normalizes the AP toward WT **(gray trace)**. **(C)** Late Na^+^ current is dramatically decreased, with a monotonic decrease back to baseline after each AP, similar to WT. **(D)** APD as a function of cycle number for 100 beats at BCL2000 corresponding to the APs shown in **B**. Note that with “boosted Mex” **(blue dots)**, there is normalization of APD similar to WT. Abbreviations as in Figures 1 and 3.

When application of boosted mexiletine was simulated in single cells, a dramatic response in the M1652R mutation was observed. Figure 5A shows the movement of the DIII-VSD during the action potential for the 3 constructs. For WT, the DIII-VSD transits between 92% and 100% “up” during an action potential. For RP, the DIII-VSD remains nearly 100% up throughout the entire duration of the action potential, underlying the sensitivity of RP to mexiletine. In contrast, the DIII-VSD of MR, even with mexiletine 10 μM, transits between 75% and 100% activated, with most of the cardiac cycle at 75% (during diastole). Application of boosted mexiletine holds “up” the DIII-VSD to ∼90% (similar to WT) and allows increased mexiletine access. In Figure 5B, the action potentials Na^+^ current and APD_90_ are plotted in response to boosted mexiletine in a fashion similar to that shown in Figure 3. Combination therapy dramatically shortened APD and late Na^+^ current and rescued the phenotype to resemble WT. This boost is further shown with the monotonic, stable APDs as a function of cycle in Figure 5B. In sum, a mexiletine booster that was designed to hold up DIII-VSD in drug-free conditions enhances mexiletine efficacy and normalizes cellular markers of arrhythmia.

**Figure 6:**
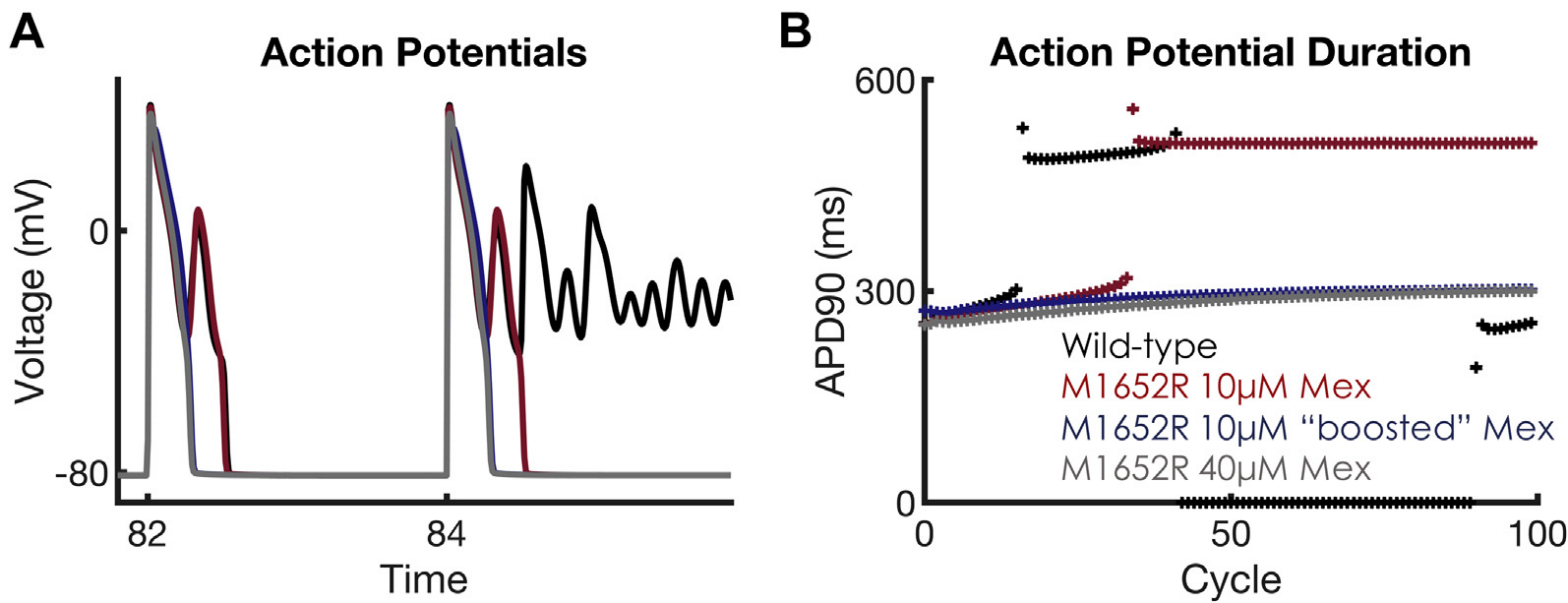
Dose-Finding Strategy for Equivalence Between Mex and Mex + Booster **(A)** Shown are APs at BCL2000, equivalent to Figure 5, at the juncture where the M1652R mutation starts the failure of the repolarization regime. After dose finding, Mex 40 μM gives APD shortening similar to that of Mex 10 μM + booster. **(B)** APD over the course of 100 cycles at BCL2000. The addition of Mex 10 μM **(red trace)**, induces repolarization at every beat, but EADs persist. This was abolished with Mex 10 μM + booster **(blue)**. To achieve equivalent APD shortening with Mex monotherapy would require Mex 40 μM **(gray)**, a 4-fold increase from the upper therapeutic range of Mex used clinically. APD_90_ = action potential duration at 90% repolarization; other abbreviations as in Figures 1 and 3.

To further characterize the efficacy of the mexiletine booster, an in silico dose-finding experiment was conducted in which we quantified the “equivalent dose” of mexiletine that would be needed to achieve comparable results with the combined booster + mexiletine 10 μM; the experiment used simulations in increments of 5 μM mexiletine, starting from the maximum therapeutic dose (10 μM). As can be seen in Figure 6, achieving similar APD shortening at BCL2000 with combination therapy (combined booster + mexiletine 10 μM) would require mexiletine 40 μM monotherapy, a 400% increase over the therapeutic limit used clinically. Thus, the use of a mexiletine booster allows increased efficacy without a concomitant increase in potentially adverse supratherapeutic dosages of mexiletine.

### 1-dimensional tissue simulations confirm the antiarrhythmic efficacy of a mexiletine booster without inducing conduction block

**Figure 7:**
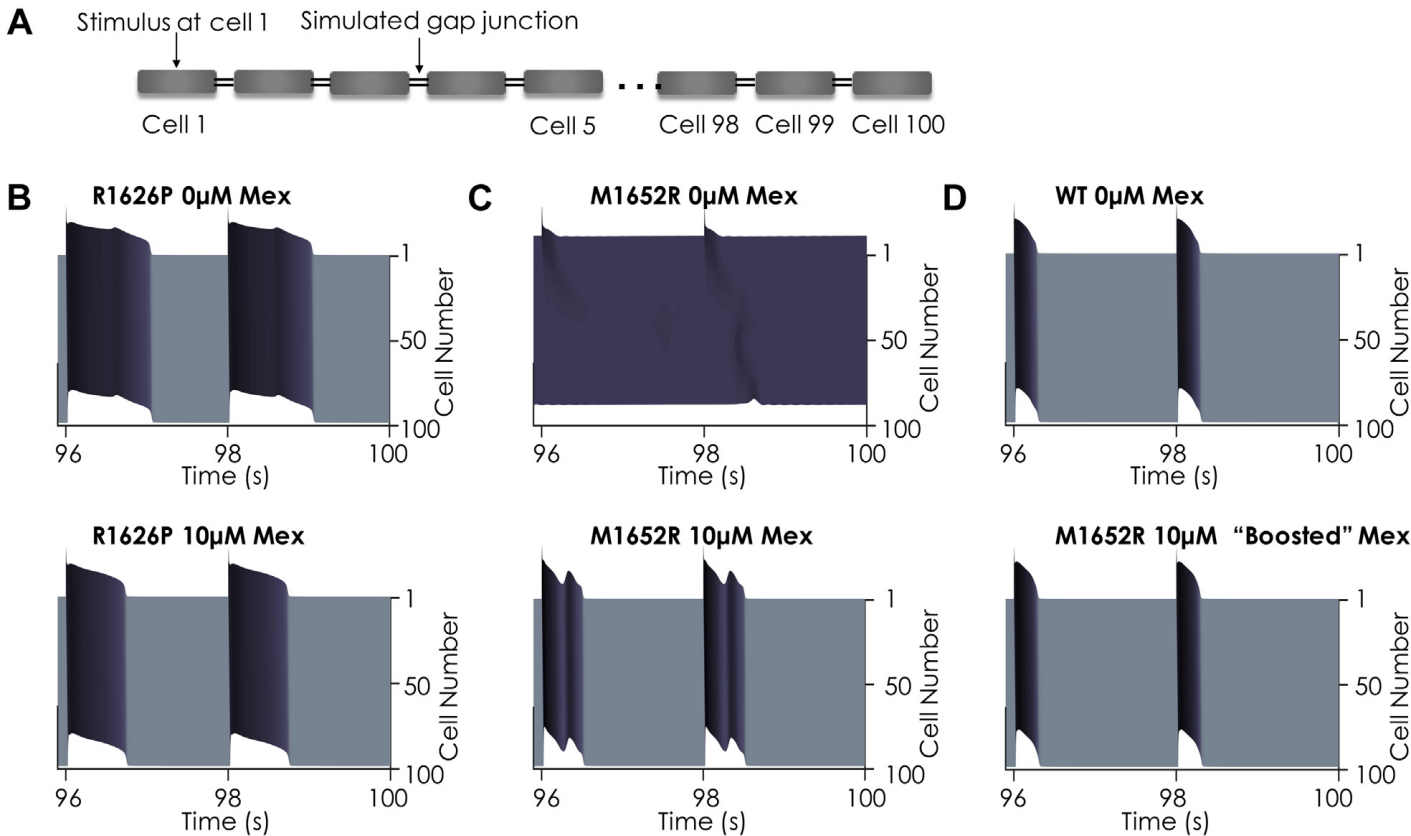
Effects of Mex on a 100-Cell 1D Cardiac Fiber Note that for all panels, time is on the x axis, cell number is on the y axis (1→100), and voltage is in the z axis. Full details of the fiber simulation are in the Supplemental Material. The last 2 beats of a 50-beat simulation at BCL2000 are shown for each condition. **(A)** Schematic of a 1-dimensional (1D) cardiac fiber. Single ventricular myocytes were electrotonically “coupled together” by simulated gap junctions. The fiber was stimulated at cell 1, and currents and transmembrane voltage were recorded for each cell in the cardiac fiber. **(B)** In the absence of Mex **(top)**, the R1626P mutation shows EADs that persist throughout fiber. Application of Mex 10 μM **(bottom)** normalizes the AP. **(C)** For the M1652R mutation in the absence of Mex, the 49th and 50th beats represent a failure of the repolarization regime, indicating sustained arrhythmia **(top)**. Mex (10 μM) repolarizes the membrane, but sustained EADs are present throughout the fiber **(bottom)**. **(D)** The WT drug-free model shows narrow APs that easily propagate throughout the fiber **(top)**. When the M1652R mutation is treated with 10 μM of “boosted” Mex **(bottom)**, the AP normalizes dramatically and resembles the WT phenotype. Abbreviations as in Figures 1 and 3.

We then simulated the effects of mexiletine alone and boosted mexiletine in a 100-cell 1-dimensional cardiac fiber. As with the single-cell results, there was sustained APD lengthening throughout a 1-dimensional simulation of a 100-cell cardiac fiber for both RP and MR. The 49th and 50th beats of a simulation at a BCL2000 were plotted. For the MR mutation, these beats are in the sustained depolarization regime. Application of mexiletine 10 μM (Figures 7B and 7C) shortened the APD for each mutant, but EADs persisted for the MR mutation. In contrast, boosted mexiletine (Figure 7D) proves efficacious in rescuing the MR mutation back to the WT phenotype.

Lastly, we assessed for conduction block by measuring conduction velocity throughout a 1-dimensional fiber using the same simulation conditions as in Figure 7 (100 cells, BCL2000, 50 beats). Within the therapeutic range of drug concentrations and pacing conditions seen clinically, mexiletine exhibited a strong degree of safety. The full analysis can be found in the Supplemental Material.

## Discussion

Despite numerous clinical failures 2, 20, antiarrhythmic drug therapy remains a cornerstone for the pharmacological management of ventricular arrhythmia. To date, all antiarrhythmic drugs are variations on a similar theme: blockade of the channel pore with varying pharmacokinetic parameters leading to varied and often unpredictable efficacy. Greer-Short et al. (21) recently reported suppression of late current in LQT3-associated mutants by narrowing intracellular cleft separation, a mechanism that depends on the local clustering of Na^+^ channels at the intercalated disc. However, given the current limitations and pressing need for novel therapeutic agents, we asked the question, can we use modulators of the channel other than the pore as novel drug targets to increase the dimensionality of the classic antiarrhythmic drugs?

To create our model, 2 unique LQT3 mutations were used as “guideposts,” given their varied clinical responses to mexiletine 8, 15, 22. These data allowed us to integrate our experimental findings into a model of the Na^+^ channel that is able to explicitly represent the molecular movements shown to be critical for mexiletine drug efficacy. To our knowledge, this analysis is the first such computational model to represent targetable molecular movements responsible for drug efficacy.

### Drug-free model development

Mathematical models to describe ion channel kinetics have proven effective at elucidating mechanisms of both normal ion channel gating as well as mutations causing aberrant channel function. State-dependent modeling has allowed for the description of the drug interaction with theoretical channel conformations, which underlie the complex kinetics and emergent behavior seen clinically 12, 23, 24. However, aside from the “open” state, all other states in previous models did not map to a specific, targetable structural correlate. The models presented here represent the next generation of kinetic modeling: combining electrophysiology kinetic data with molecular-level structural insight 10, 17.

We focused on the DIII-VSD for a number of reasons. It is becoming increasingly clear that the 4 VSDs can exhibit subtle dynamic changes and cause a bidirectional effect on pore conformation. Previous studies showed that lidocaine, a Class Ib antiarrhythmic drug binding to the pore, affects DIII-VSD dynamics 7, 25, and we previously found that LQT3 variants can alter DIII-VSD activation; DI, DII, and DIV seem to play less of a role (8). Thus, we focused our efforts at incorporating DIII-VSD movement into a kinetic-based model of the Na^+^ channel.

We began by using 2 LQT3 mutations to parameterize our kinetic model: R1626P, a mexiletine-sensitive mutation, and M1652R, a mexiletine-resistant mutation, in addition to wild type. When mexiletine was given to a carrier of M1652R, there was no change in QT interval, and the patient subsequently developed sudden cardiac death (ventricular fibrillation). In contrast, a carrier of the R1626P mutation exhibited a 12.5% reduction in QT interval and has remained alive with no cardiac events after 7 years of follow-up at the time of publication (15). Thus, these 2 mutants served as a natural starting point. After numerical optimization, the final models incorporated a wealth of electrophysiological data that captured current kinetics over a wide range of pacing protocols, as well as fluorescent data that tracked the DIII-VSD molecular movement in response to changes in membrane voltage.

### Mexiletine drug blockade

We then expanded these models to account for mexiletine drug blockade, with representations for drug access derived from the modulated and guarded receptor hypotheses 26, 27, 28, microscopic reversibility (29), DIII-VSD results (8), and clinical effects of mexiletine 15, 22. Our constraint requiring equivalent affinity of mexiletine to the local anesthetic receptor for each mutation, verified experimentally by us (8) and others (15), ensured that the observed differences in the model simulations were a direct result of DIII-VSD movement. The cellular and tissue simulations recapitulated the effects seen clinically: therapeutic doses of mexiletine failed to normalize the marked APD prolongation and emergent EAD triggers seen in the M1652R mutation. In the extended time course for the M1652R mutation (Supplemental Figure 4), chaotic behavior was noted, with salvos of sustained membrane depolarization preceded by increasing APD, strikingly similar to the 2:1 atrioventricular block as well as sudden cardiac death/ventricular fibrillation at the whole heart level seen clinically (15). Conversely, the R1626P mutation exhibited a 28% reduction in APD_90_ from baseline with mexiletine 10 μM, similar to clinical results.

We chose to simulate slow pacing frequencies, given that LQT3 arrhythmia syndromes are bradycardia dependent, happening mostly during sleep and periods of inactivity (30). Often, they are much less pronounced during normal (and fast) heart rates, given the rate-dependent QT shortening. This rate dependence can best be seen in Supplemental Figure 9: both mutations clearly display pathologic QT prolongation (as seen clinically [15]). Based on our simulations, the M1652R displays marked variation in rate-dependent APD, with severe prolongation only at bradycardic pacing. The important overall distinction, however, is that M1652R remains resistant to therapeutic mexiletine (Figure 3), whereas R1626P can effectively be treated with current therapies even at tachycardic pacing (dashed blue traces in Supplemental Figure 9 represent mexiletine 10 μM).

Based on our modeling, we found that R1626P significantly prolonged the QT interval even more than M1652R in single-cell simulations. Furthermore, Figure 3F shows that mid-dose mexiletine (5 μM) was not particularly effective for the R1626P mutation, which displays beat-to-beat variability in APD. However, we found no simulations that suggested further degeneration of this variability (e.g., sustained failure of repolarization for example), as was seen with M1652R (Supplemental Figure 4). Simulating a clinical approach, we found that we could overcome this chaotic behavior by applying high-dose mexiletine (10 μM) with R1626P. Whether beat-to-beat variability versus EADs play more of a proarrhythmic role is still unclear clinically, although these mechanisms are likely a continuum of similar phenomena (e.g., EADs can lead to beat-to-beat variability and vice versa).

### Rational design of mexiletine booster

We then sought to leverage computational modeling to rationally design a precision-targeted, molecularly based therapy to enhance mexiletine. The data explicitly reveal 3 positions of the DIII-VSD and suggest a fourth: 1) the rested position (R), in which DIII-VSD is in a “down” position, drug is inhibited from receptor access, and the DIII-VSD is maximally fluorescent; 2) the first activated state (A1), in which the DIII-VSD has moved progressively “up” in the membrane and fluorescence starts to quench, but the receptor is still blocked; 3) the second activated state (A2), in which DIII is fully “up” and fluorescence is maximally quenched, and which allows for drug binding; and 4) DIII-VSD is fully “up” but the channel has shifted to a kinetically inactivated regime. This fourth regime is based on our biotin experiment (Figure 4), as well as our previous studies 8, 18, which strongly suggest that the DIII-VSD plays a critical role in channel inactivation.

Our approach to designing an in silico mexiletine booster was therefore to optimize the drug-free rate constants responsible for DIII-VSD movement in the MR mutation to simulate the hyperpolarizing shift in SSA of MR back to WT, and a 2-fold increase in tonic block and UDB. Simply by changing the rates governing the drug-free DIII-VSD movement, we were able to simulate the effects of “holding up” DIII-VSD on drug binding. In our computational simulations (Figure 5A), the mexiletine booster effectively “trapped” DIII-VSD in the up position throughout the action potential and revealed a crucial result: a relatively small change in DIII-VSD fluorescence at resting membrane potential (75% vs. 90% with the mexiletine booster) had a dramatic effect on mexiletine’s clinical efficacy.

### Clinical utility and implications

Mexiletine remains widely prescribed, most commonly for malignant ventricular tachycardia. In contrast to flecainide, it can be used in both structurally normal and abnormal, infarcted hearts. It is often used as an oral lidocaine analogue in those patients who derived clinical benefit from intravenous lidocaine. It is preferred over amiodarone for long-term management of ventricular arrhythmia, given the long-term consequences of chronic amiodarone therapy (pulmonary, liver, thyroid, and cornea deposition). Interestingly, mexiletine has also been used to treat neuropathic pain (31). Although widely used, tolerance to mexiletine is inversely proportional to the dose, with gastrointestinal distress and nausea often limiting high therapeutic concentrations.

As a necessary first step and proof-of-concept, we focused on 2 LQT3 mutations that represent the “extremes” of DIII-VSD sensitivity to mexiletine. By framing the modeling study in terms of these 2 mutations, however, we were able to delineate the putative molecular mechanism that underlies Na_V_1.5 sensitivity to mexiletine, regardless of the presence or absence of a mutation. In our previous study (8), we further found a strong correlation between DIII-VSD activation and mexiletine sensitivity (Figure 4 and Online Table II in the paper by Zhu et al. [8]). We also found that WT channels are not particularly sensitive to mexiletine, obviating high doses of mexiletine used clinically with increased side effects for modest therapeutic benefit. Thus, it is conceivable that the booster strategy could work for WT channels implicated in varied cardiac arrhythmia syndromes (e.g., ventricular tachycardia in which mexiletine is commonly used) by further hyperpolarizing the WT DIII-VSD activation voltage, which would result in increased WT sensitivity.

Drug development remains a markedly expensive and time-consuming endeavor fraught with potential failure throughout the development cycle. The computational modeling approach presented here may be a novel strategy to increase the chances of success by rationally designing drugs in silico while simultaneously assessing markers of success and failure at multiple spatial and time scales. The strength of computational modeling is highlighted by a few key results; namely, we were able to rationally design a drug molecule based on key molecular movements of the Na^+^ channel, and then test those predictions in coupled higher dimensional tissue. Second, we were able to perform an in silico safety analysis to assess for hallmark proarrhythmic sequelae of Class I drugs, namely conduction block. Finally, we were able to use the model to quantify the strength of our mexiletine booster through an “equivalence” dose-finding simulation. Rather than de novo drug design of a new antiarrhythmic drug, our model suggests a potential polypharmaceutical strategy to use 2 drugs with synergistic effects: a commonly prescribed antiarrhythmic drug that accesses the local anesthetic drug receptor on the Na^+^ channel combined with a DIII-VSD modulator for increased antiarrhythmic efficacy. As shown in the state-dependent drug-binding analysis of Supplemental Figures 3 and 4, a relatively small increase in DIII-VSD movement can have a dramatic effect on therapeutic doses of mexiletine.

This “boosted” approach, or pharmacokinetic enhancement, has been used previously with notable examples, including human immunodeficiency virus antiretroviral agents, drugs for Parkinson’s disease, and cancer immunotherapy. The addition of a “booster,” in this case an allosteric modulator, combined with a pore blocker adds an entirely new dimension to the existing parameter space of antiarrhythmic drug efficacy. Although we tested this approach with LQT3 and mexiletine, we expect that a similar strategy is widely applicable to those with malignant and refractory ventricular tachycardia both from ischemic heart failure and nonischemic, inherited arrhythmia syndromes. We further expect that boosting other Class I drugs could yield similar benefit.

### Study limitations

Although the Na^+^ channel model is highly complex and is coupled with a highly parameterized human ventricular myocyte computational model, it represents a simplification of the true underlying pathophysiology. The molecular movements include only the contribution from the DIII-VSD, as our experimental results (8) suggest that this domain is the most important for determining Class I antiarrhythmic drug efficacy. Future studies may incorporate the contribution of the DIV-VSD as a next step. As noted, all current Class I drugs are designed to have different binding rates and affinities to the channel pore; a “booster” allows for the modulation of the state of the channel. However, it is possible that activation of DIII-VSD may be proarrhythmic. Based on our simulations, our in silico booster molecule seems to be extremely effective as an adjunct therapeutic strategy with mexiletine and could be a clinically useful therapy. However, such a molecule does not currently exist, and in fact, despite the existence of DIV-VSD–binding molecules, we are aware of no DIII-VSD molecule currently in clinical development. We further note that we used a saturating concentration of biotin, and our simulations of “booster” necessarily pushed the DIII-VSD into a nearly complete “upward” position to fit the desired higher affinity tonic and use-dependent block. Theoretically, one could pursue further dose-finding strategies to “fine-tune” the desired therapeutic effects of the “booster” molecule. Although a booster does not exist currently, this study provides a rationale to pursue such a molecule.

## Conclusions

We have developed a model of the Na^+^ channel that for the first time includes explicit representation of the molecular movements which shape the action potential and form the basis of the differential sensitivity to the common antiarrhythmic drug mexiletine. After expanding the model to account for the pharmacokinetic variables of mexiletine drug binding and the role of the DIII-VSD, the model was used to develop a precision-targeted mexiletine booster that was able to effectively rescue a mexiletine-resistant LQT3 mutation. Our results suggest a promising future avenue of drug development, namely exploitation of nontraditional ion channel drug targets, which allows for precision-targeted and mutation-specific pharmacotherapy for both LQT3 mutations and an enhanced mexiletine for ventricular tachycardia, with a more favorable side effect profile.

Perspectives **COMPETENCY IN MEDICAL KNOWLEDGE:** Class I antiarrhythmic therapies remain suboptimal given the inability to predict efficacy and potential proarrhythmic side effects. The Class Ib drug mexiletine has been show to have differential efficacy based on key molecular determinants of the Na+ channel. This study used computational modeling to understand the molecular basis of drug efficacy, and then designed an in-silico mexiletine booster that could improve efficacy.

**TRANSLATIONAL OUTLOOK:** Drug discovery remains expensive, time-consuming, and has an unacceptably high failure rate. Computational modeling approaches to drug discovery represent a novel tool to design and test precision-targeted therapeutic agents, premised on a detailed understanding of the molecular underpinnings of drug efficacy. By exploiting nontraditional ion channel drug targets, we can add an entirely new dimension to the wide parameter space of traditional antiarrhythmic drugs to develop more precision-targeted and potent Class I therapeutic agents.
