# Supplementary material for: A Molecularly Detailed NaV1.5 Model Reveals a New Class I Antiarrhythmic Target
Source: JACC Basic Transl Sci. 2019 Oct 28;4(6):736–51. doi: 10.1016/j.jacbts.2019.06.002 (PMC6834944; doi:10.1016/j.jacbts.2019.06.002)
Supplement: Supplemental Data [file mmc1.pdf]

A molecularly detailed Nav1.5 model reveals a new class I antiarrhythmic target

Jonathan D. Moreno, MD, PhD; Wandi Zhu, BS; Kathryn Mangold, MS; Woenho Chung, BS; Jonathan R. Silva, PhD

SUPPLEMENTARY INFORMATION

Materials and Methods Summary

A computational Markov model of the WT, M1652R, and R1626P LQT3 mutants were formulated with and without the mexiletine drug channel interaction via numerical optimization from experimental data as described previously [1, 2] that includes both channel kinetics and voltage-camp fluorescence describing the DIII-VSD movement. The drug channel models were incorporated into a computational model of the human ventricular myocyte [3] to assess cellular and tissue response to drug therapy. All simulations, numerical optimization and data visualization were done in MATLAB 2017A™. Detailed methods are below. Source code is available for download.

Drug-free models

The wild-type drug free Na^+ channel model previously published [4] was used as the basis of the present study. Briefly, the original model included 8 states: 3 closed states (C3, C2, C1), an open state (O), 2 closed-inactivated states (IC3, IC2), a fast-inactivated state (IF), and a slow inactivated state (IS). The model accurately simulates kinetics of physiological gating including activation, inactivation (closed and open state), recovery from inactivation (fast and slow), and channel mean open time. Further details can be found in [1, 4].

The model was expanded to account for DIII-VSD movement. Our experimental results suggest at least 3 conformations of the DIII-VSD: resting, partially activated, and fully activated. This was modeled as 8 additional states; 4 denoted with the prefix (R) – resting, and 4 denoted (A1) – partially activated. The 8 top states from the previous model are modeled as having DIII fully activated (A2). These 16 states are necessary to capture both the current kinetics, as well as fluorescent kinetics of activation and inactivation.

Three different models were optimized using numerical optimization methods as detailed below; a wild-type drug free model (WT), and two LQT3 mutant models (R1626P, M1652R). Each model was fit to multiple voltage clamp and fluorescent protocols to constrain model behavior. These included steady-state availability (SSA), steady state activation (ACT), recovery from inactivation (RFI), deactivation (TAU), fluorescent activation (FLU ACT), fluorescent tau (FLU TAU), late current (LATE), and A1/A2 fluorescent partition. The wild-type model was further constrained by channel mean open time at -30mV (MOT).

The protocols were as follows:

- ***Steady-state availability (SSA):*** For each voltage between -125mV to -30mV, the steady-state probabilities of the channel were found. The channel was then pulsed to -10mV, and the open-state probability was determined. The value of the open state probability was then normalized to the open state probability at -125mV.
- ***Steady-state activation (ACT):*** Channel steady-state was found at -160mV. The channel was then stepped to voltages between -160 – 20mV, and for each voltage, the channel maximum open probability was calculated, and the conductance GNa was determined. Values were then normalized to GNa at 20mV.
- ***Fluorescent activation (FLU ACT):*** Channel steady-state was found at -160mV. The channel was then stepped to voltages between -160 – 20mV. Channel fluorescence was determined after a 100ms step to the test potential. Fluorescence was calculated as the

sum (A1 + A2 states) / total states. Fluorescence was normalized to the total fluorescence calculated at +20mV.

- ***Tau of deactivation (TAU):*** Channel steady-state was found at a holding potential of -120mV. From steady-state, the channel was then stepped from -80mV to +20mV, and the Tau of 50% decay of the channel current was calculated for each voltage.
- ***Fluorescent tau (FLU TAU):*** From a steady-state of -120mV, the channel was then pulsed to potentials between -80mV to +20mV for 40 seconds. Channel fluorescence was then calculated (peak fluorescence). The channel was stepped back to -120mV, and the decay of 50% fluorescence from the peak was calculated. Fluorescence was calculated as before: sum (A1+A2 states) / total state occupancy.
- ***Recovery from inactivation (RFI):*** From a steady-state of -100mV, the channel was then pulsed to +20mV, and peak current was found. The channel was then allowed to recover for a variable time, before being repulsed to +20mV. The test current peak was then normalized to the initial peak current.
- ***Late current (LATE):*** From a steady-state of -100mV, the channel was pulsed to -10mV for 400ms, before repolarization to -100mV for 2600ms (total BCL3000ms). After 10 pulses, the late current (measured after 400ms depolarization of the 10th pulse) was normalized to the peak current after the 10th pulse.
- ***A1/A2 Fluorescent partition:*** Based on the curve of Figure S5, the relative ratios of the A1 fluorescence (A1C3, A1C2, A1C1, A1C4) and A2 fluorescence (A2C3, A2C2, A2C1, A2O, A2OS, A2IC3, A2IC2, A2IF) were calculated.
- ***Mean open time (MOT):*** At -30mV, the WT channel was optimized to have a mean open time of 0.5ms[5]. This was calculated as the reciprocal of the sum of the states leaving the open state: MOT = 1/(a2 + b13 + ay + a5).
- ***Tonic block (TB):*** From a holding potential of -100mV at steady-state, the channel was pulsed to -10mV; TB was measured as the amount of block that occurred after the first pulse to -10mV, normalized to drug-free first pulse peak current.
- ***Use-dependent block (UDB):*** From a holding potential of -100mV at steady-state, the channel was pulsed to -10mV for 400ms, followed by repolarization back to -100mV for 100ms (to simulate the depolarization time for a normal human action potential). UDB was calculated as the peak current after 100 pulses normalized to the peak current after the first pulse at each drug concentration.

Drug-bound mexiletine models

The 16-state drug free model of each construct (WT, MR, RP), was expanded to account for mexiletine drug binding. As detailed in our previous modeling studies [4], drug modeling was based on assumptions for drug access derived from the modulated and guarded receptor hypotheses, and included pH dependent partitioning of charged and neutral fractions, as well as the clinical effects of Na^+ channel blocking drugs. To that end, each of the top 12 drug free states (note, no drug binding to the 4 rested states), had a corresponding charged drug and neutral drug bound state. Thus, each overall model consisted of 40 states (16 drug-free, 12 charged drug-bound, and 12 neutral drug-bound).

For both charged and neutral drug species, we assume that drug can only access the receptor when the DIII is in the fully activated conformation (A2 states). However, once bound, the channel could enter into the partially activated states (A1). As noted above, resting DIII (DIII in the “down” position) impairs access of mexiletine to the receptor, and thus, there are no drug bound rested states.

The model parameters for the drug binding rates for mexiletine were derived from experiments or computed as described below. These included diffusion rates that indicate drug on rates "k_{on}" = [drug]*Diffusion and affinities to discrete conformations that determine drug off rates "k_{off}" = Kd*Diffusion. Given similar pKa of mexiletine to flecainide, diffusion was assumed similar to flecainide [6], and a value of 5000 M^{-1} ms^{-1} was used in the model. Rates were constrained by experimental data (described in detail below) and by implementing microscopic reversibility as in Colquhoun [7].

As previously detailed, we assume that similar to flecainide and lidocaine, the charged fraction of mexiletine does not readily access inactivated states, and can only bind when the channel is in A2C3, A2C2, A2C1, or A2O. Open state affinity for the charged form of mexiletine was derived from Kd values obtained from experiments measuring use-dependent blocking (UDB) affinity, an estimate of affinity to the open state [5]. This affinity was defined as Kd0 – the Kd at 0 mV. Closed state affinity of charged drug was then calculated using Eyring rate theory for the voltage dependence of rate constants ($Kd = Kd0 * e^{(-d*V*F/(R*T))}$) [8], where F, R, and T have their usual biophysical significance, and d is the fractional electrical distance of the blocking site (0.7)[9]. After simultaneous optimization of the LQT drug-models, the Kd0 value was found to be 156μM. The computed Kd value at -100 mV for mexiletine was 2449μM, an estimate of the charged drug affinity for the closed state, and similar to experiment (~1000μM)[10].

The state specific affinities of neutral mexiletine to inactivated states were taken from Ruan et al.[10] which derived a theoretical affinity to the inactivate states as 13μM; we used this in our model. No experimental data exists for the neutral fraction of mexiletine to the closed and open states; we chose a value of 1000μM which is in the range of experimental values for flecainide and lidocaine [9, 11]. Both of these affinities are not voltage dependent, nor allowed to change during the optimization. As compared to mexiletine, neutral mexiletine can bind directly to the inactivated states; thus, there is binding from A2IC3 to D_A2IC3. The transition arrows are omitted in Figure S1 for clarity but denoted by the blue box surrounding those states.

Each drug block model was constrained by the following voltage and fluorescent protocols as described above: steady state availability with 250μM Mex (SSA), tonic-block (TB) and use-dependent block (UDB) (1 – 1000μM Mex), recovery from inactivation with 250μM Mex (RFI), fluorescent activation with 4000μM Mex (FLU ACT), fluorescent deactivation at 0mV with 4000μM Mex (FLU TAU), and late current blockade with 75μM Mex.

Numerical optimization procedure

All computations were done in MATLAB 2017B. We coded all voltage and fluorescent protocols and used the matrix exponential technique as described in [2] for simulation. A modified Nelder Mead Simplex Method that allowed for constrained optimization (only positive rate constants) was used for simultaneous optimization of the protocols specific to each model. A cost function for each protocol was defined as the sum of squared differences between experiment and simulation. The total cost function (sum of the individual protocol errors) was then minimized and converged when a tolerance of 0.01 for the change of the cost function and 0.01 for the change in parameters was achieved. Further details can be found in [1]. Each drug-free model was optimized separately, given their independence. In contrast, because the drug block models required parallel optimization of charged drug affinity, the LQT models (RP, MR) were optimized simultaneously. The optimization routine first optimized the drug rate constants for the MR and RP mutation simultaneously to determine the intrinsic affinity of charged mexiletine to the receptor at 0 mV (Kd_0 = 156μM). The wild-type model drug rates were then optimized while holding Kd_0 constant.

Cellular and cardiac fiber simulations
The model formulation for virtual cardiac cells was from Grandi et. al.[3], with the Na^+ channel replaced with the model described here. LQT mutations were considered to be "heterozygote", with 50% current coming from the LQT mutant Na^+ channel formulation and 50% coming from WT Na^+ channel formulation. WT simulations were 100% WT Na^+ channel formulation. The maximal conductance of the Na^+ channels were set to give realistic peak current density and the largest current still yielding full repolarization at BCL1000ms, as well as physiologically comparable conduction velocities in a 1D fiber (see Figure S8). Thus, GNa_{WT} = 65 mS/cm^2, GNa_{RP} = 1000 mS/cm^2, GNa_{MR} = 6.5 mS/cm^2. These yield peak Na^+ currents of -302pA/pF (WT), -152 pA/pF (MR), and -90pA/pF (RP). All baseline parameters were used from the published Grandi-Bers model, except the Cl- background current maximal conductance, which was increased from 9e-3 to 13.5e-3 mS/ μF for enhanced repolarization. The stiff solver ODE15S (MATLAB) with a variable time step was used for numerical integration of the system of ODEs.

Single cells were electrotonically "coupled" together to create a 100-cell virtual cardiac fiber. A diffusion term implemented, and the differential equation system was solved with the operator-splitting method of Qu and Garfinkel [12]. The cardiac fiber was "paced" (application of current) at cell 1 (100pA/pF) for the first 5ms of each beat, and action potentials were recorded at each cell. We chose to use a diffusion coefficient of D = 0.00154cm^2/ms, as used previously by us [4, 13] and others [14], which gives realistic conduction velocities in 1-dimensional coupled tissue.

Molecular biology
Electrophysiology recordings
Mutations were introduced in the human Na_V1.5 α subunit carried in the pmax vector with overlap extension PCR and In-fusion cloning (Clonetech). After mutagenesis is confirmed with sequencing (Genewiz), mutant NaV1.5 α subunits are co-transfected with Na_V β1 subunit into HEK 293T cells with jetPRIME transfection kit (Polyplus transfection). Na^+ currents were measured using whole-cell patch clamp configuration with Axopatch 200B amplifiers (Axon instrument). Glass pipettes with access resistance of 3-4 MΩ and 80-90% serial resistance compensation were used for recording. I-V protocol was measured by stepping to a series of testing potentials ranging from -120 to 40 mV from a holding potential of -120mV. Steady state inactivation (SSI) protocol was executed by testing channel current availability with a -20mV testing pulse, after holding cells at various holding potentials ranging from -150 to 0mV for 200ms.

A note about concentrations of mexiletine used in the study. Cell expression systems tend to be much less sensitive to drug than in-vivo studies for a few reasons. Most importantly, experiments are done at a much lower temperature (~22C) to capture the electrophysiologic kinetics, which would be too fast at 37C. Second, cell expression systems lack the physiologic milieu, which makes direct comparison of concentrations difficult. We have also found in our previous study [15] that the concentration dependence is *highly* cell expression system specific. In that study, we found that the cut-open voltage clamp had a 2-3 fold higher EC_{50} as compared to the two-electrode voltage-clamp (TEVC) and patch clam setup for oocytes due to presumably obstructed access to solutions during perfusion (see Online Table I [15]).

We further found that the TEVC technique gave comparable results to those obtained in HEK cells, which we used in this current manuscript wherever possible. Given these limitations, we have tried to reconcile a number of data sources and have tried to be as explicit as possible in this manuscript. Given the translational aspect of our work, we found it important to limit the drug concentrations simulated in the cellular and tissue studies (simulation of human physiology) to those concentrations used clinically. Thus, 10μM mexiletine was the highest concentration tested in our simulations, to correlate with the maximum therapeutic concentration used clinically.

MTSEA-Biotin Experiment

Channel modification by external MTSEA-biotin was tested with previously described protocols [16]. MTSEA-biotin was reconstituted in DMSO to a stock concentration of 1mM, which is then diluted in external recording solution to a final concentration of 2µM. After MTSEA-biotin's binding to the R1306C channel stabilizes (~20 minutes after perfusion), current properties were assessed again with IV and SSI voltage protocols. Then the same cell was perfused with external solution containing 100µM mexiletine to test block. For control group, mexiletine block was tested with external solution containing 100µM mexiletine.

HEK-293 transformation of M1652R steady-state availability (SSA)

Simultaneous recording of VCF and cell electrophysiology currently utilizes the *Xenopus* oocyte cell expression system. While this system is robust, it lacks potential modifying factors in Na_V1.5 cell expression. For example, recording of M1652R in *Xenopus* oocytes fails to recapitulate the ~15mV depolarizing shift in SSA as seen by us [15] and others [10] in the more physiologic HEK-293 cell expression system. We have determined that this depolarization is crucial in understanding the variation between M1652R, R1626P and WT. To that end, we fit our SSA curve of M1652R obtained in *Xenopus* oocytes to a standard Boltzmann function to obtain a $V_{1/2}$ of -66mV. We then induced a 15mV depolarizing shift and fit the computational model with the assumption of a $V_{1/2}$ of -51mV (-66mV + 15mV), similar to previously published results of M1652R in HEK-293 cells [10]. We kept k, the slope factor, the same. Thus, in Figures 1 and 2, the red circles represent the non-transformed data, the red triangles represent the transformed data that the model was fit to, and the solid red line represents the optimized model.

Voltage Clamp Fluorometry

Voltage-clamp fluorometry (VCF) is a technique that allows simultaneous observation of changes in channel conformation and ionic current kinetics. Briefly, fluorophore tethering is typically accomplished by introducing a cysteine residue into an area of interest and labeling it with a thiol-reactive fluorophore. To track VSD conformation, the fluorophore is usually conjugated to a cysteine in the S3-S4 linker [15]. A previously developed construct of DIII (M1296C) was used for VCF recordings. mRNA of the NaV1.5 channel constructs was coinjected with the NaV β1 subunit in Xenopus oocytes. Voltage clamp recordings were performed 4 to 5 days after injection. The recording setup, solutions, and recording protocols for VCF are the same as described previously [17-19].

Statistics

Results for experimental data were expressed as mean ± standard error of the mean (SEM). Significances between groups were tested using the Student's t-test.

Model parameters

Drug-free rate constants

Rate	***WT***	***M1652R***	***R1626P***
a11_variable1	1.2575e-02;	7.5606e-02;	6.1216e-02;
a11_variable2	8.8239e+01;	1.1430e+02;	2.5341e+02;
a12	3.8372e-01;	2.2108e+00;	1.8514e+00;
a13	5.6577e-01;	2.3181e+00;	1.1394e-01;
b11_variable1	4.5755e+00;	1.3341e+01;	5.7543e+00;
b11_variable2	7.9064e+00;	7.9443e+00;	1.0572e+01;
b12	6.4541e-01;	2.3436e+00;	5.2469e+00;
b13	4.4474e+00;	7.8042e+00;	4.9374e+00;
a3_variable1	1.0018e-03;	1.0004e-03;	1.1346e-03;
a3_variable2	1.2672e+01;	1.0928e+01;	1.4444e+01;
b3_variable1	1.5264e+01;	6.5872e-01;	1.9902e+01;
b3_variable2	1.6025e+01;	3.0722e+01;	2.9843e+01;
a2_variable1	8.6189e+00;	9.8118e+00;	1.2276e+01;
a2_variable2	6.4138e+02;	9.9996e+02;	1.8536e+02;
a5	3.4869e+00;	7.6692e-02;	2.5849e+00;
b5	1.5977e-01;	1.4339e-01;	1.1137e-02;
ax_variable1	3.4592e-01;	1.4491e+00;	1.8563e-01;
ax_variable2	3.1104e+01;	2.5459e+01;	2.6972e+01;
bx_variable1	2.6028e+01;	6.8111e+01;	3.8993e+01;
bx_variable2	2.4524e+02;	6.0932e+02;	4.8885e+02;
ay_variable1	2.7321e-03;	3.2828e-03;	1.1597e-03;
ay_variable2	2.0203e+01;	2.2526e+01;	1.9490e+01;
by_variable1	1.7737e+01;	3.5316e+01;	2.9943e+00;
by_variable2	1.9338e+01;	1.4246e+01;	6.6924e+01;

Where the transition rates are of the form:

```
a11= Tfactor*1/(Input_DF(1)*exp(-V/Input_DF(2)));
a12= Input_DF(3)*a11;
a13= Input_DF(4)*a11;
b11= Tfactor*1/(Input_DF(5)*exp(V/Input_DF(6)));
b12= Input_DF(7)*b11;
b13= Input_DF(8)*b11;
a3 = Inputs_BTN(1)*Tfactor*Input_DF(9)*exp(-V/Input_DF(10));
b3=  Inputs_BTN(2)*Tfactor*Input_DF(11)*exp((V)/Input_DF(12));
a2=  Tfactor*(Input_DF(13)*exp(V/Input_DF(14)));
b2=  ((a13*a2*a3)/(b13*b3));
a5 = Input_DF(15) *a2;
b5 = Input_DF(16) *a3;
ax = Inputs_BTN(3)* Tfactor*(   Input_DF(17)*exp(-V/Input_DF(18))    );
bx = Inputs_BTN(4)* Tfactor*(   Input_DF(19)*exp(V/Input_DF(20))   );
ay = Inputs_BTN(5)* Tfactor*(   Input_DF(21)*exp(-V/Input_DF(22))    );
by = Inputs_BTN(6)* Tfactor*(   Input_DF(23)*exp(V/Input_DF(24))  );

Q10=3; T = 295; Tfactor = 1.0/(Q10^( (37.0-(T-273))/10.0));
```

Note: **b2** is constrained by microscopic reversibility.

In-silico Biotin rates

Rate	***Biotin rate (M1652R only)***
Input_BTN(1) – a3 transition modification<br>Input_BTN(2) – b3 transition modification<br>Input_BTN(3) – ax transition modification<br>Input_BTN(4) – bx transition modification<br>Input_BTN(5) – ay transition modification<br>Input_BTN(6) – by transition modification	1.0259e-01;<br>4.2013e-01;<br>5.0451e+01;<br>3.4531e+00;<br>3.4669e-02;<br>1.7154e+00;
Where the transition rates are of the form:<br>a3 = Inputs_BTN(1)* Tfactor*Input_DF(9)*exp(-V/Input_DF(10));<br>b3 = Inputs_BTN(2)* Tfactor*Input_DF(11)*exp((V)/Input_DF(12));<br>ax = Inputs_BTN(3)* Tfactor*(Input_DF(17)*exp(-V/Input_DF(18)));<br>bx = Inputs_BTN(4)* Tfactor*(Input_DF(19)*exp(V/Input_DF(20)));<br>ay = Inputs_BTN(5)* Tfactor*(Input_DF(21)*exp(-V/Input_DF(22)));<br>by = Inputs_BTN(6)* Tfactor*(Input_DF(23)*exp(V/Input_DF(24)));<br><br>Q10=3; T = 295; Tfactor = 1.0/(Q10^((37.0-(T-273))/10.0));	

Note, biotin rates are only used for the M1652R mutation. When simulating wild-type and R1626P, an Inputs_BTN vector of all 1's should be used. (Inputs_BTN = [1;1;1;1;1;1]). This vector of 1's should also be used when simulating the M1652R mutation in the absence of the mexiletine booster. See the included Matlab code and the manuscript for details.

Affinities and other drug binding parameters

Pharmacokinetics and biologics

```
pH=7.4;
pKa=9.2;
portion = 1.0/(1+ 10^(pH-pKa));
diffusion= 5000;
drug=Drug*(1e-6);
drug_charged=drug*portion;
drug_neutral=drug*(1-portion);
dd= -0.7;
R = 8314.472;
F = 96485.3415;
T = 295;
```

Charged drug binding kinetics

```
kd0=156.0*(1e-6);
kd_open=kd0*exp( (dd*V*F) /(R*T));

kon=drug_charged*diffusion;
koff=kd_open*diffusion;
kcon = kon;
kcoff = koff;
```

Neutral drug binding kinetics

```
k_on = drug_neutral*diffusion;
k_off=1000.0*(1e-6)*diffusion;

ki_on = drug_neutral*diffusion;
ki_off = 13.0*(1e-6)*diffusion;
kc_on = k_on;
kc_off = k_off;
```

Mexiletine drug rate constants

Rate	*WT*	*M1652R*	*R1626P*
ay1_variable1	3.8280e-03;	3.4309e-04;	4.4524e+01;
ay1_variable2	1.2009e+01;	9.0788e+01;	1.0227e+02;
by1_variable1	1.7711e+02;	6.1486e+01;	1.0497e+01;
by1_variable2	2.2736e+01;	7.1355e+00;	1.9847e+02;
a13c	2.8246e+02;	2.1363e+02;	2.8761e+01;
a22	3.5949e+02;	4.9057e-02;	1.8831e-01;
a33	1.1670e-04;	6.7711e-02;	4.5401e-01;
b33	1.3495e-03;	2.4495e-02;	1.2438e+01;
a55	1.0575e+02;	1.8059e-02;	8.5769e-01;
b55	1.3132e-02;	2.4511e-04;	5.5022e-03;
ay2	5.6878e+02;	1.1068e+01;	7.0782e+02;
by2	1.0895e+02;	1.2122e-01;	7.7915e+02;
a13n	1.5115e+02;	4.4038e+02;	3.9201e+00;
b_33	2.4327e+00;	1.4203e+01;	3.7494e-01;
a_22	1.6249e+00;	9.8467e+02;	9.7842e+02;
a_55	3.6865e+02;	5.8506e+02;	8.4655e+02;

Where the transition rates are of the form:

```
ay1 = Tfactor*(   Inputs_Drg(1)*exp(-V/Inputs_Drg(2))    );
by1 = Tfactor*(   Inputs_Drg(3)*exp(V/Inputs_Drg(4))   );
a13c = Inputs_Drg(5)*a13;
a22 = Inputs_Drg(6)*a2;
a33 = Inputs_Drg(7)*a3;
b33 = Inputs_Drg(8)*b3;
a55 = Inputs_Drg(9)*a5;
b55 = Inputs_Drg(10)*b5;
ay2 = Inputs_Drg(11)*ax;
by2 = Inputs_Drg(12)*bx;
a13n = Inputs_Drg(13)*a13;
b_33 = Inputs_Drg(14)*b33;
a_22 = Inputs_Drg(15)*a2;
a_55 = Inputs_Drg(16)*a5;

Q10=3; T = 295; Tfactor = 1.0/(Q10^( (37.0-(T-273))/10.0));
```

Note: **b13c, b22** are constrained by microscopic reversibility (charged drug regime), and **b13n, a_33, b_22, b_55** are constrained by microscopic reversibility (neutral drug regime).

Of note, in the Matlab script, the drug free model of each construct contains 24 parameters, noted as the “drug-free rate constants”. These are input as a matrix “Input_DF” (DF is “drug-free”. For example, Input_DF (1) corresponds to a11_variable1, and Input_DF(2) corresponds to a11_variable2, Input_DF(3) corresponds to a12, and Input_DF(4) corresponds to a13 etc. The transition rate constants are of the form denoted with the “Input_DF” matrix. In similar fashion, each drug-bound mexiletine model contains 16 parameters (e.g. ay1_variable1, ay1_variable2, by1_variable1 etc.), and correspond to the matrix “Inputs_Drg”, noted in the transition rates in the above tables.

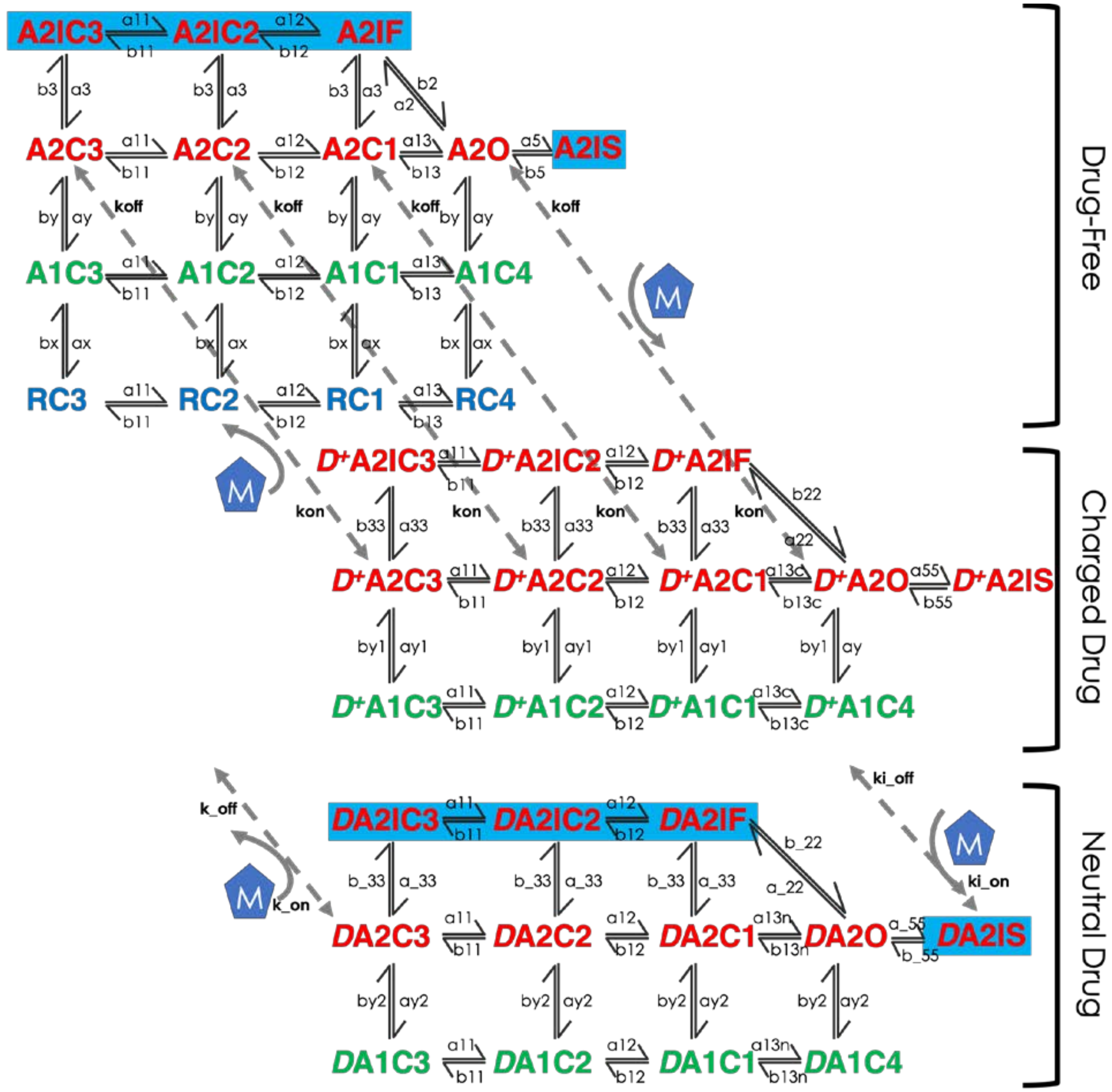

Figure S1: Model schematic of mexiletine drug binding

Markovian model representation of the drug Na^+ channel interaction. The drug free channel has 16 distinct states with 3 regimes of DIII-VSD: rested (blue), first activated (green) and second activated (red). The A1 and A2 regimes can exist as drug-bound states, but mexiletine can only access the receptor from the A2 states. There are two modes of drug bound channel states: the middle of the schematic represents entry or egress from charged (denoted D^+) drug bound states, and the bottom third represents entry or egress from neutral drug bound states. Gating transitions that occur subsequent to drug binding may be affected by presence of drug. See **Materials and Methods** for details. Transition arrows were omitted from A2IC3←→DA2IC3 (neutral), A2IC2←→DA2IC2 (neutral), A2IF←→DA2F (neutral), and A2IS←→DA2IS (neutral) for clarity (blue box). Rate constants listed in the Figure can be found in the **Supplementary Material**.

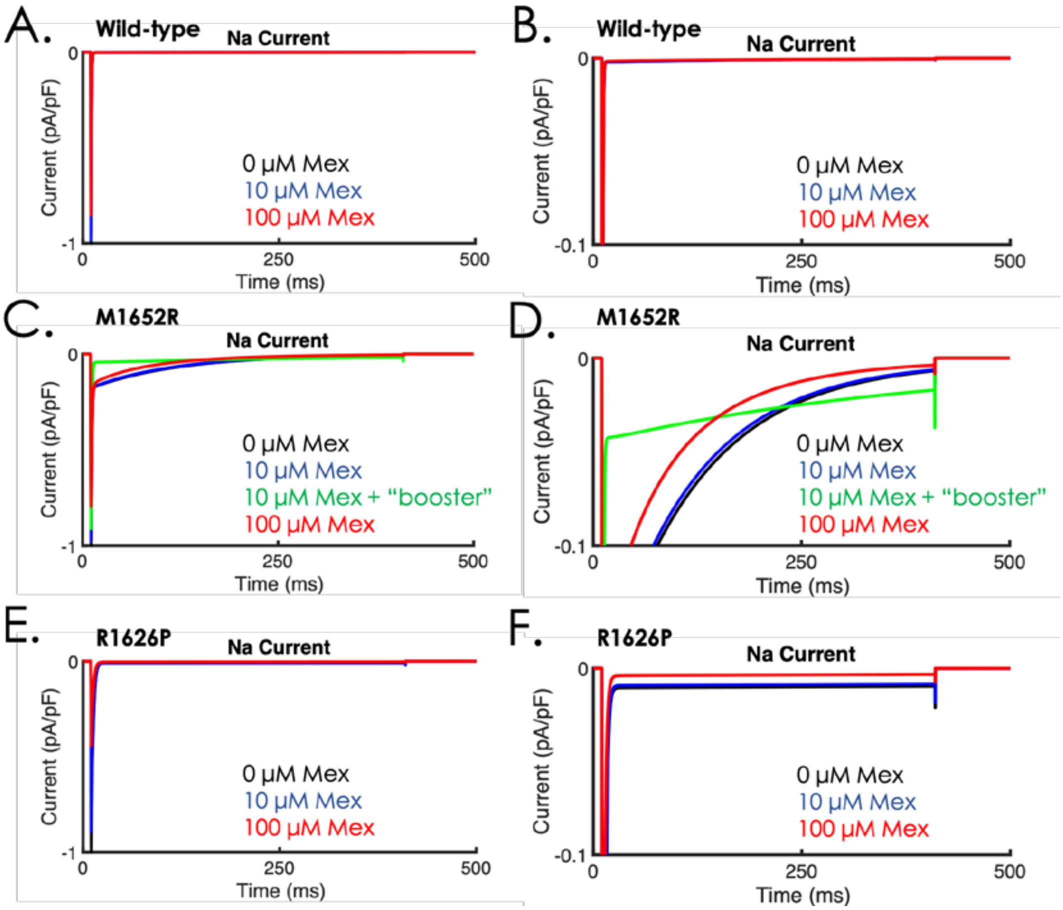

Table: Peak and late current normalized to drug free conditions

	0μM Peak (Raw)	0μM Late (Raw)	10μM Peak	10μM Late	100μM Peak	100μM Late
**WT**	100% (236.4 pA/pF)	0% (-0.12 pA/pF)	98%	0%	85%	0%
**M1652R**	100% (-613.4 pA/pF)	0.67% (-4.119 pA/pF)	97%	0.62%	79%	0.37%
**R1626P**	100% (-6.4 pA/pF)	1.0% (-0.0617 pA/pF)	89%	0.8%	44%	0.32%

Figure S2: Na^+ currents in response to mexiletine at BCL3000ms.
From a steady-state of -100mV, the channel was pulsed to -10mV for 400ms, before repolarization to -100mV for 2600ms (total basic-cycle length (BCL) of 3000ms), to more closely resemble the time course of the human ventricular action potential. After 10 pulses, the late current (measured after 400ms depolarization of the 10th pulse) was normalized to the peak current after the 10th pulse. Of note, this protocol is identical to the LATE protocol used in the optimization routine to optimize late currents. The traces are each normalized to the drug-free (0μM Mex) condition (black traces). The raw current magnitude for the drug-free conditions are noted in parentheses in the table above. As can be seen, with 10μM mexiletine (blue traces), there is minimal effect on the peak inward Na^+ current (WT – 98%, M1652R – 97%, RP – 89% current remaining); approximately the same amount of blockade is seen for the late current at this drug concentration (e.g. 0.62% current remaining for M1652R vs. 0.67% current in the drug free

condition). Panel D, a zoomed in view of M1652R, shows that 10μM mexiletine + “booster” dramatically decreases the contribution of the late current throughout the duration of the action potential, underlying its therapeutic efficacy. Panels B and F show the corresponding zoomed in view for WT, and R1626P, respectively.

With regards to the “boosted” mexiletine in Figure 4G; the differences in late blockade with 75μM are grossly similar (36% vs 31% with “boosted” mexiletine). This is primarily a result of the design of the “booster” to reflect a shift in SSA, and 2-fold increased sensitivity to both TB, and UDB only. As a result, the booster had little effect on the absolute magnitude of the late current in our simulations (see table below). This is not entirely unexpected, as the rate constants governing slow recovery (primarily responsible for the late current) were not allowed to change during the “boosted” mexiletine optimization process.

	Peak current (pA/pF)	Late current at 200ms (pA/pF)	% late blockade
Drug-Free M1652R	-613.4	-19.6	--
M1652R, 75uM Mex	-364.8	-12.5	36%
M1652R, 0uM Mex, with “booster”	-569.4	-17.7	--
M1652R, 75uM Mex, with “booster”	-232.8	-12.2	31%

In sum, the “booster” as is simulated and “designed” currently, has a more appreciable effect on peak current blockade (responsible for TB and UDB). It would be an interesting avenue of future research to design an in-silico booster that has selectivity for the late current.

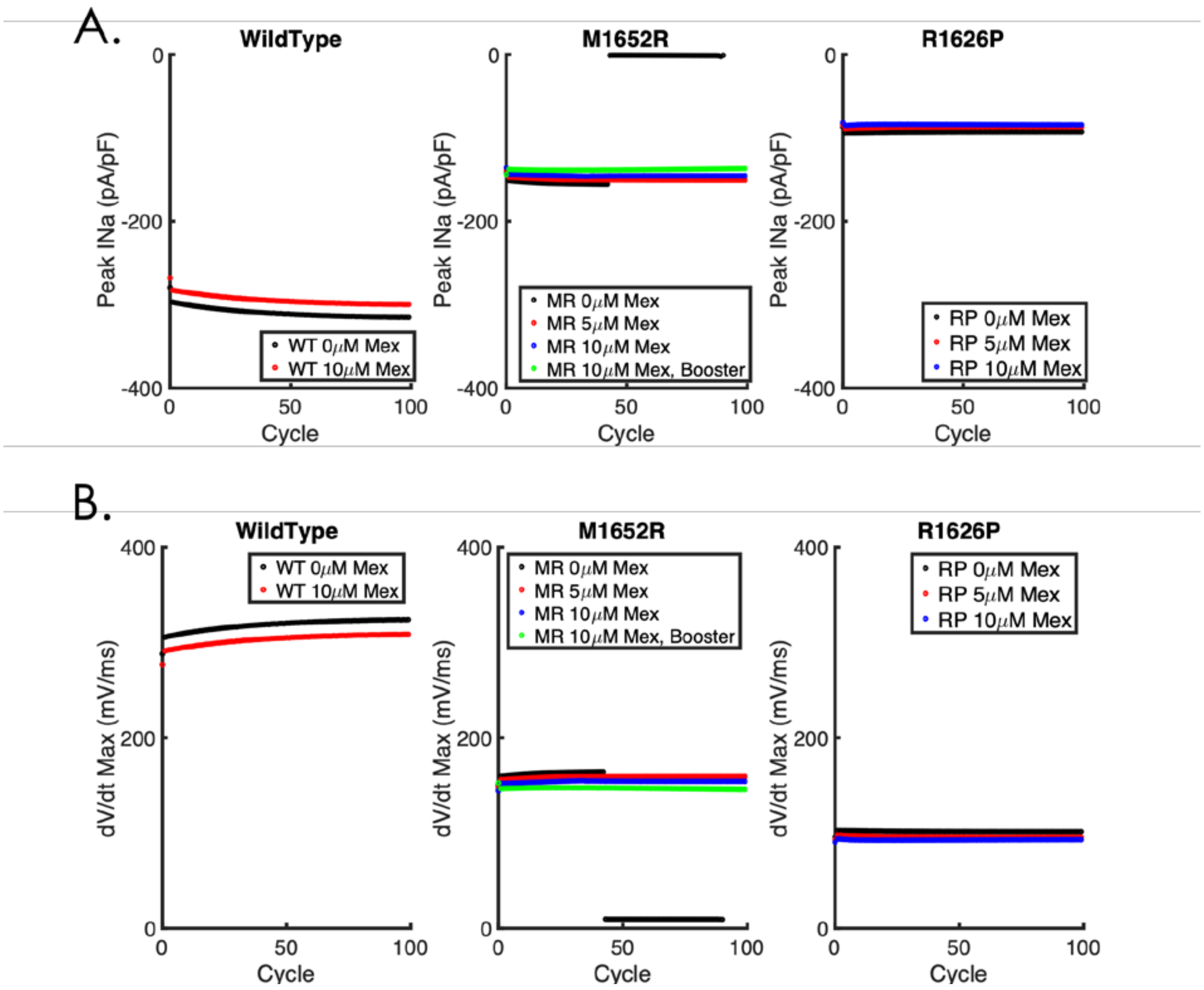

Figure S3: Cellular properties and mexiletine drug blockade

Figure 3 of the manuscript depicts cellular action potentials, late Na^+ current, and action potential duration in response to escalating doses of mexiletine. In the Figure above, peak I_{Na} (panel A) as well as maximum upstroke velocity (dV/dt_{MAX} – panel B) is plotted as a function of cycle for the same bradycardic pacing regime shown in Figure 3 (BCL 2000ms). Note that within the therapeutic concentration range tested for mexiletine (0 - 10μM), there is minimal decrement of both peak Na^+ current as well as maximum upstroke velocity as compared to the drug-free condition of each mutation (compare black trace 0μM vs. blue trace 10μM). Notably, R1626P, owing to a large leftward (hyperpolarizing) shift in steady-state availability has markedly decreased peak I_{Na} and dV/dt_{Max}. As shown in Figure 6, this does ***not*** lead to higher dimensional conduction blockade within the therapeutic range of mexiletine. As seen in the cellular action potentials (Figure 3), there is a regime of failed repolarization (sustained depolarization) in the drug-free M1652R condition (regime where peak I_{Na} and dV/dt_{Max} = 0 above). Action potential repolarization is restored for each action potential upon application of mexiletine $\pm$ "booster". Importantly within the drug concentrations tested, there is no failure to capture of any action potential, a single cell marker of proarrhythmic conduction block.

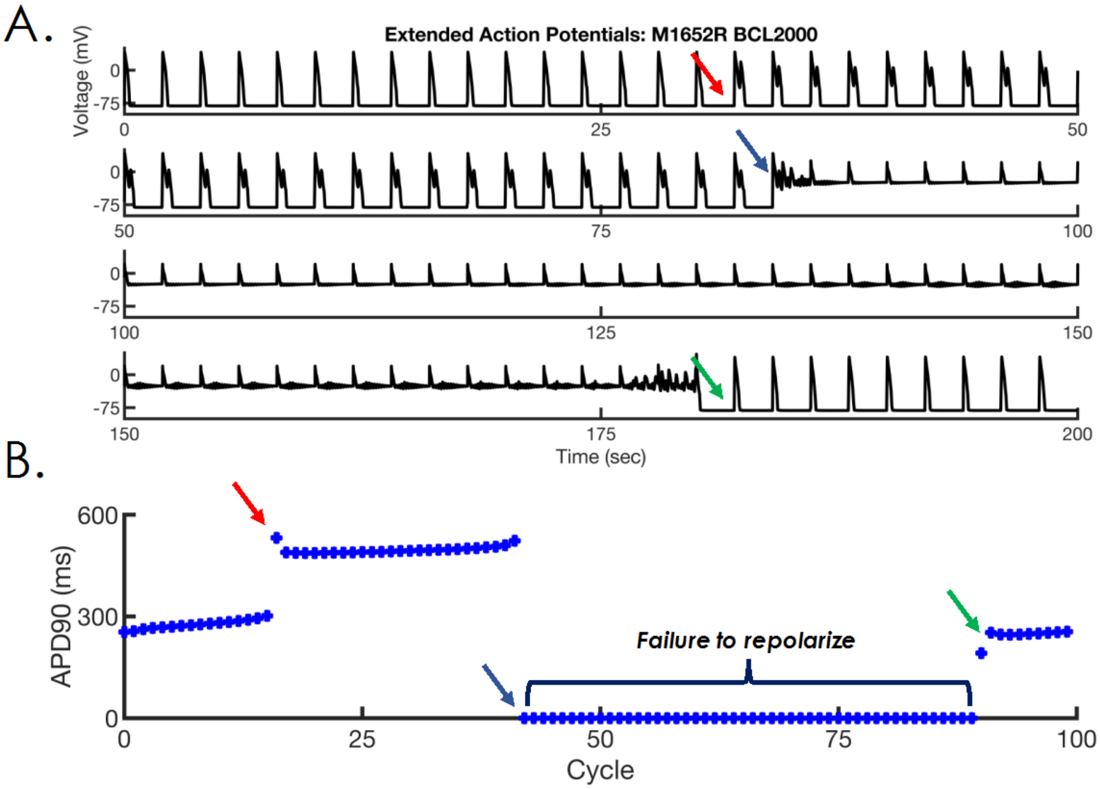

Figure S4: Extended time course of cellular action potentials at BCL2000 for M1652R.

A. Action potentials for all 100 paced beats is shown (extended simulation of Figure 4). Note the onset of EADs at beat 17 (red arrow). The AP becomes progressively longer until failure to repolarize (blue arrow). At beat 90, the cell finally repolarizes to a normal AP (beat 91, green arrow).
B. APD is shown or all 100 paced beats. The colored arrows correspond to the individual APs depicted in panel (A): onset of EADs (red arrow), failure to repolarize (blue arrow), onset of repolarization (green arrow). The simulation plots the failure to repolarize regime as an APD = 0ms (blue arrow to green arrow).

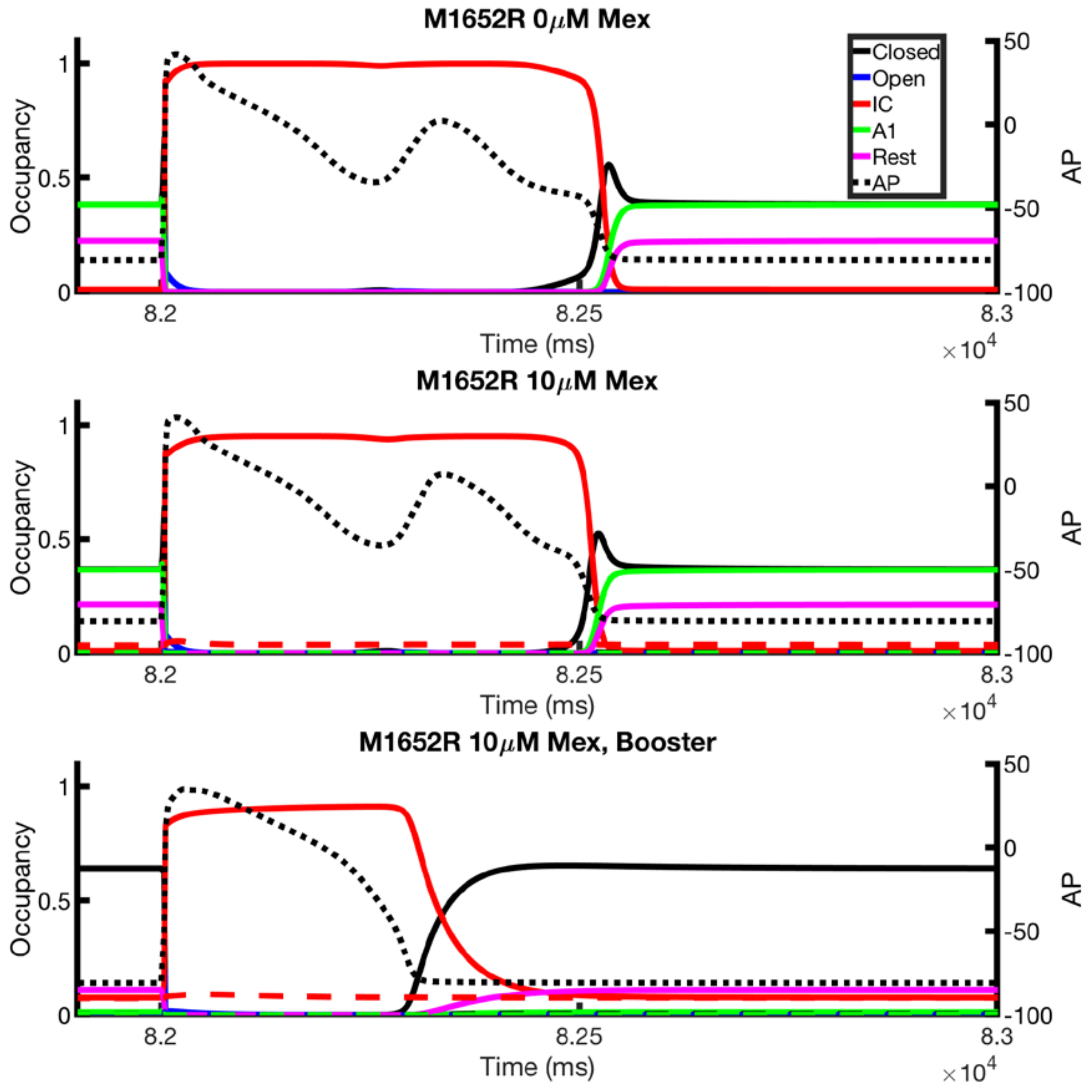

Figure S5: State occupancies of the drug free regime
Shown above are the state occupancies of the 16 drug-free states of the model in the absence of mexiletine (top), 10μM mexiletine (middle), and 10μM "boosted" mexiletine (bottom). Overlaid are APs (dashed lines). At resting membrane potential (RMP), in both 0μM and 10μM mexiletine, approximately 21% of the channel is in the resting regime (pink), 36% is in the A1 regime (green), and 36% is in the A2C regime (black). After the initial upstroke of the AP, the majority of the channels quickly inactivate (red trace), before recovering to steady state conditions at RMP. With "boosted" mexiletine (bottom), the relative proportion of channel residences change: 10% are in the resting regime (pink), virtually none are in A1 (green) and the majority (64%) are now in the A2C regime (black), as well as A2IC regimes (7%, red). Membrane depolarization again pushes channels to the inactivated states (red) before returning to steady state. Note, however, the absence of the EAD seen in the top two panels.

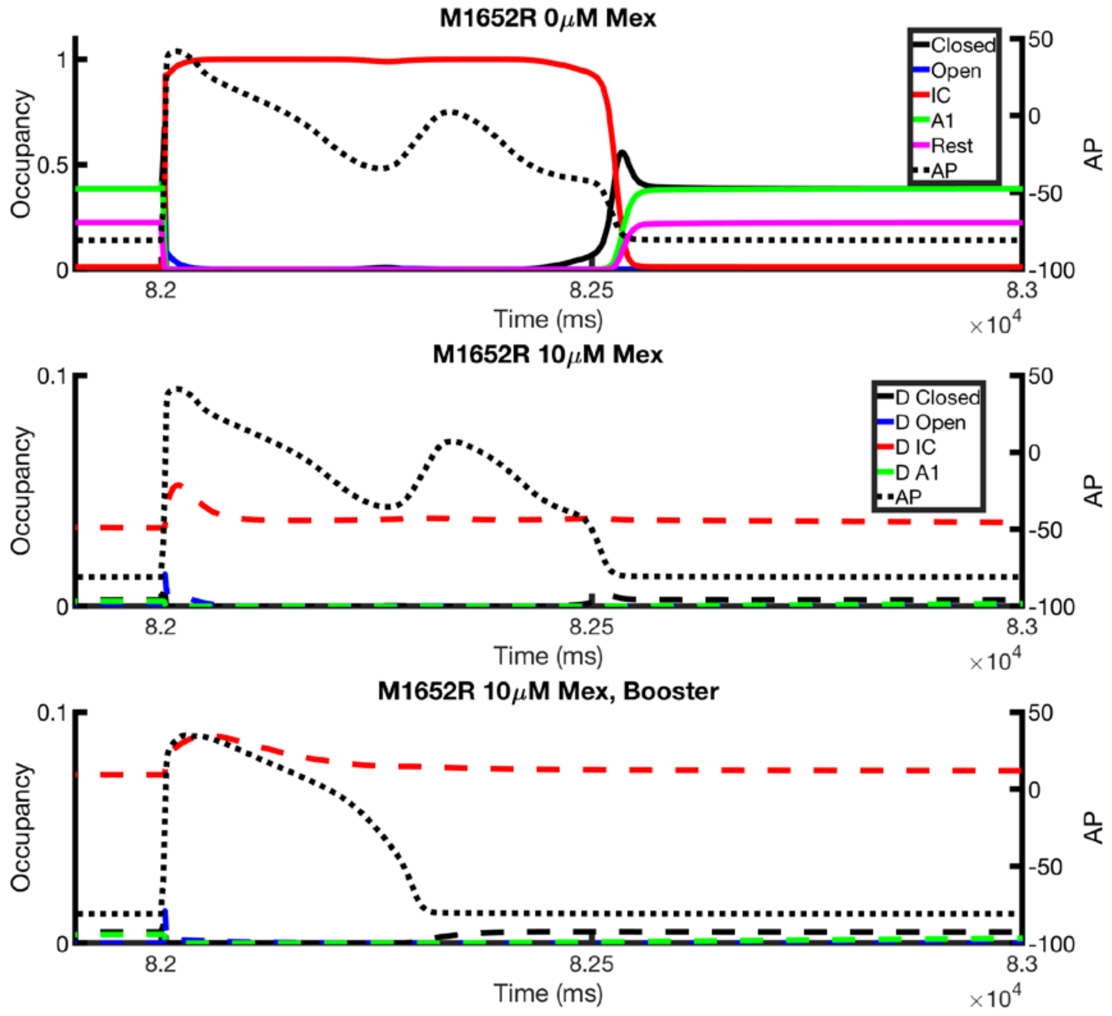

Figure S6: State occupancies of the drug bound regime

A similar analysis as depicted in Figure S3 is shown for the drug bound regime. The addition of the mexiletine “booster” leads to more effective mexiletine drug binding. Analysis of the drug bound states shows that stabilization of the drug-bound inactivated states is important for drug efficacy (dashed red curve in bottom two panels). In the absence of the “booster” (middle panel), about 3% of the channels are in the drug-bound inactivated states at RMP; with “boosted mexiletine, the proportion doubles to ~7.5% (bottom panel), which is enough to normalize APD as shown in the overlaid action potential (black dashed line).

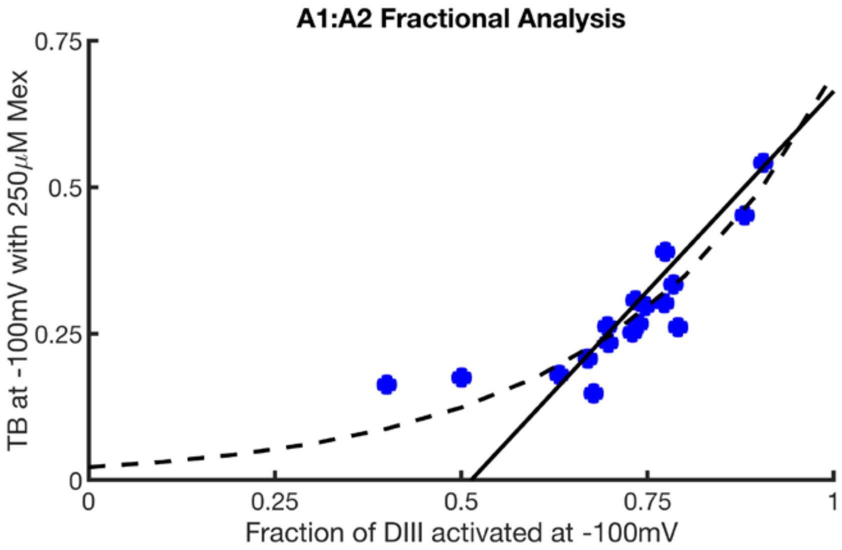

Figure S7: Analysis of DIII-VSD fluorescence between A1 and A2
The above plot represents the relationship between TB and fraction of DIII activated at -100mV (drug-free). There is a saturating effect when the DIII-VSD is less than ~50% activated, indicating a low level of DIII-VSD independent block (likely representing hydrophobic, lipophilic block). The relationship becomes linear within the 70% – 90% DIII-VSD activation range. We used this relationship to derive the fractional A1:A2 ratio as follows:

1) We assume that for mexiletine to bind, the DIII-VSD must be in the fully upright (A2) position. As noted in the manuscript, drug binding only happens from the A2 states.

2) We create a line of best fit (solid line above). The resultant equation is y = 1.3636x -0.7. The x-intercept is thus 0.5133, which is equal to the initial fluorescence when DIII-VSD makes the A1 →A2 transition. Thus, a fluorescence value below 0.5133 is assumed to be from the A1 states. We can then assume that the total range of DIII-VSD in the A2 position is between x = 0.5133 and 1.0 (a range of 0.4867). Stated another way, at x = 0.5133, no DIII-VSDs are in the A2 position, whereas at x = 1, 100% of DIII-VSDs are in the A2 position.

3) At -100mV, in the absence of drug, WT = 71.2% DIII-VSD activated, RP = 92.6% DIII-VSD activated, and MR = 50% DIII-VSD activated (e.g. x values of the mutants above). We can then calculate the ratio of A2 as follows:

- WT: (0.712 – 0.5133) / 0.4867 = 41% in A2
- RP: (0.926 – 0.5133) / 0.4867 = 84% in A2
- MR: (0.50 – 0.5133) / 0.4867 = 0% in A2

As an experimental validation, we can calculate an intrinsic affinity of mexiletine to the receptor when we assume DIII-VSD is in a 100% “up” position (x = 1). At x = 1, y = 0.6636 (66.36% block) with 250μM mexiletine. Assuming a simple binding scheme we can calculate an EC_{50} value:

$$33.64\% = \frac{1}{1 + \frac{250\mu M}{EC_{50}}}$$

The EC_{50} value is 126.9μM, which is remarkably similar to the 156.9μM from the optimized model.

Figure S8: Analysis of conduction velocity and block in a 1-Dimensional Cardiac Fiber

Conduction Velocity at BCL2000ms			
	**Wild-type**	**M1652R**	**R1626P**
**0μM Mex**	122.5cm/s	*failed repolarization	111.6cm/s
**10μM Mex**	122.5cm/s	104.2cm/s	97.7cm/s
**10μM Mex + "booster"**	--	104.2cm/s	--
Conduction Velocity at BCL500ms			
**0μM Mex**	122.5cm/s	97.7cm/s	89.3cm/s *(3:2 block)
**10μM Mex**	122.5cm/s	91.9cm/s	89.3cm/s *(2:1 block)
**25μM Mex**	122.5cm/s	82.2cm/s	66.5cm/s
**50μM Mex**	122.5cm/s	69.4cm/s	***CB**
*Conduction velocity was calculated as the difference in time of initial deflection from resting membrane potential between cell 1 and 50, as measured on the 50th beat of a 50 beat, 100-cell simulation at either BCL2000ms or BCL500ms. As noted, conduction velocity of M1652R drug free could not be calculated as there was global failure to repolarize throughout the tissue. In the simulations dx = 0.025cm; thus the time differential was the time required to conduct through 1.25cm of tissue (0.025cm *50 cells).			

As opposed to flecainide (class IC), mexiletine is a class IB drug, with relatively fast on/off kinetics which spares peak I_{Na}. It exerts its antiarrhythmic effects primarily by decreasing the effective refractory period. When we simulated our *in-silico* mexiletine booster in single cells, we were able to dramatically rescue the MR disease phenotype; APD_{90} was normalized to resemble WT, EAD arrhythmia triggers were abolished, and no beat-to-beat chaotic behavior was noted (see Figure 5). However, conduction block and reentry is an emergent phenomena only seen in higher dimensions and represents a fundamental proarrhythmic mechanism of class I drugs.

To asses for conduction block, we measured conduction velocity (CV) throughout a 1-dimensional cardiac fiber using the same simulation conditions as Figure 7 (100 cell, BCL 2000ms, 50 beats). As can be seen in Table 1 and predicted from the concentration-dependent blocking isotherms in Figure 2B (use-dependent block) and 2C (tonic block), 10μM mexiletine has relatively little effect on conduction velocity. Under drug-free conditions, the WT 1D cardiac fiber has a CV of 122.5cm/s. M1652R CV drug-free cannot be accurately assessed at this same timepoint, given multiple regimes of failure of repolarization. However, both 10μM mexiletine, as well as 10μM mexiletine + "booster", maintain robust CV of 104.2cm/s. R1626P in the drug-free and 10μM mexiletine condition have CVs of 111.6cm/s, and 97.7cm/s, respectively. This relative slowing of CV is secondary to the large leftward shift of steady-state availability curve, as well as increased therapeutic efficacy at 10μM mexiletine for this mutation. Importantly, however, despite this large leftward shift and increased sensitivity, all APs propagate throughout the 1D fiber without conduction block, indicating a high degree of safety with this drug concentration.

We then undertook the same analysis at the "extreme": tachycardic pacing at BCL 500ms. For both the WT, and M1652R mutation, no conduction block is exhibited even at supratherapeutic mexiletine (50μM). Owing to a large leftward shift in steady-state availability, the R1626P mutation does indeed exhibit conduction block at 50μM (5x the upper therapeutic range of mexiletine). Interestingly, therapeutic levels of mexiletine enhance repolarization as compared to drug-free conditions, such that 25μM mexiletine allows for all action potentials to propagate, as compared to drug-free (3:2 block, given failed repolarization), and 10μM (2:1 block). In sum, within the physiologic range of drug concentrations and pacing regimes tested, mexiletine with or without

booster does not dramatically affect peak I_{Na} and lead to conduction block in higher dimensions. This gives us confidence that the model recapitulates the strong degree of safety of conduction exhibited with class 1B antiarrhythmic drugs (e.g. preserved conduction) [4, 20].

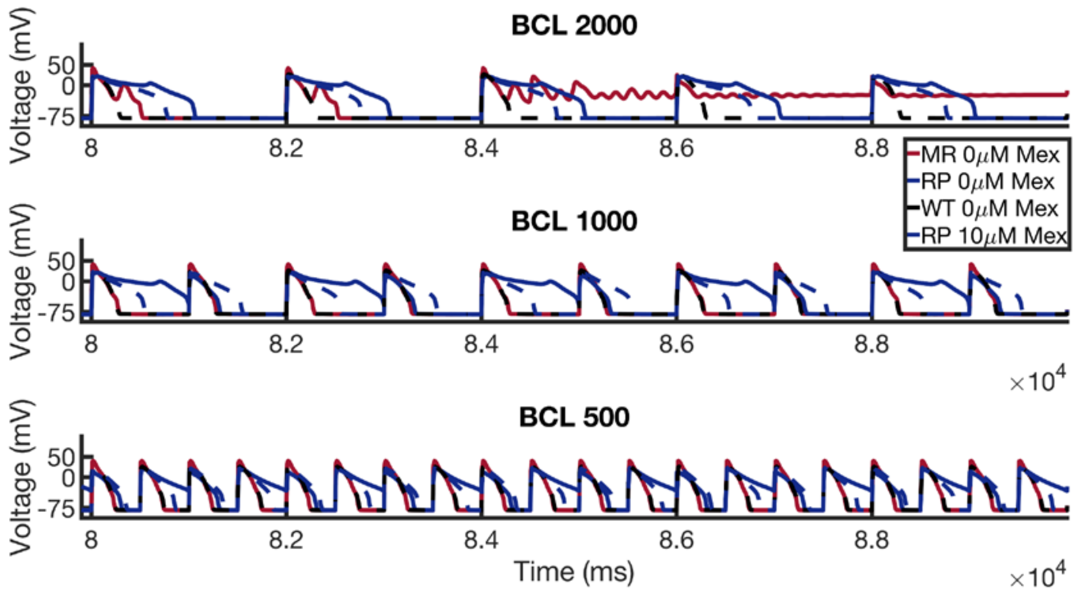

Figure S9: Rate-dependence of LQT3 mutations and mexiletine

We undertook an additional analysis to show the rate dependence of the two LQT3 mutations tested across a wide pacing regime. As noted in the manuscript, we chose to simulate slow pacing frequencies, given that LQT3 arrhythmia syndromes are bradycardia-dependent, happening mostly during sleep and periods of inactivity [21]. Often, they are much less pronounced during normal (and fast) heart-rates, given the rate-dependent QT shortening. As seen in the Figure above, both mutations clearly display pathologic QT prolongation (as seen clinically [10]) at bradycardic pacing (BCL 2000ms). Application of 10μM mexiletine with M1652R (Figure 3) fails to normalize EAD triggers, whereas for R1626P, application of 10μM mexiletine (dashed blue traces above) exhibits moderate APD reduction. At BCL1000ms, the M1652R mutation shows a marked reduction in APD (red trace), similar to WT (dashed black trace). Conversely, R1626P remains prolonged throughout all pacing regimes, with episodes of "R on T" phenomena (note BCL1000ms solid blue line at 81, 83, 85 sec – the onset of the next AP happens as the previous AP is repolarizing). The important overall distinction, however, is that M1652R remains resistant to therapeutic mexiletine (Figure 3), while R1626P can effectively be treated with current therapies even at tachycardic pacing (see dashed blue traces in all 3 plots above: R1626P with 10μM mexiletine).

REFERENCES

1. Moreno, J.D., T.J. Lewis, and C.E. Clancy, *Parameterization for In-Silico Modeling of Ion Channel Interactions with Drugs.* PLoS One, 2016. **11**(3): p. e0150761.
2. Teed, Z.R. and J.R. Silva, *A computationally efficient algorithm for fitting ion channel parameters.* MethodsX, 2016. **3**: p. 577-588.
3. Grandi, E., F.S. Pasqualini, and D.M. Bers, *A novel computational model of the human ventricular action potential and Ca transient.* J Mol Cell Cardiol, 2010. **48**(1): p. 112-21.
4. Moreno, J.D., Z.I. Zhu, P.C. Yang, J.R. Bankston, M.T. Jeng, C. Kang, L. Wang, J.D. Bayer, D.J. Christini, N.A. Trayanova, C.M. Ripplinger, R.S. Kass, and C.E. Clancy, *A computational model to predict the effects of class I anti-arrhythmic drugs on ventricular rhythms.* Sci Transl Med, 2011. **3**(98): p. 98ra83.
5. Liu, H., M. Tateyama, C. Clancy, H. Abriel, and R. Kass, *Channel Openings Are Necessary but not Sufficient for Use-dependent Block of Cardiac Na+ Channels by Flecainide Evidence from the Analysis of Disease-linked Mutations.* Journal of General Physiology, 2002. **120**(1): p. 39-51.
6. Zhu, Y., J.W. Kyle, and P.J. Lee, *Flecainide sensitivity of a Na channel long QT mutation shows an open-channel blocking mechanism for use-dependent block.* Am J Physiol Heart Circ Physiol, 2006. **291**(1): p. H29-37.
7. Colquhoun, D., K.A. Dowsland, M. Beato, and A.J. Plested, *How to impose microscopic reversibility in complex reaction mechanisms.* Biophys J, 2004. **86**(6): p. 3510-8.
8. Yue, D.T., J.H. Lawrence, and E. Marban, *Two molecular transitions influence cardiac sodium channel gating.* Science, 1989. **244**(4902): p. 349-52.
9. Bennett, P.B., C. Valenzuela, L.Q. Chen, and R.G. Kallen, *On the molecular nature of the lidocaine receptor of cardiac Na+ channels. Modification of block by alterations in the alpha-subunit III-IV interdomain.* Circ Res, 1995. **77**(3): p. 584-92.
10. Ruan, Y., N. Liu, R. Bloise, C. Napolitano, and S.G. Priori, *Gating properties of SCN5A mutations and the response to mexiletine in long-QT syndrome type 3 patients.* Circulation, 2007. **116**(10): p. 1137-44.
11. Liu, H., J. Atkins, and R.S. Kass, *Common molecular determinants of flecainide and lidocaine block of heart Na+ channels: evidence from experiments with neutral and quaternary flecainide analogues.* J Gen Physiol, 2003. **121**(3): p. 199-214.
12. Qu, Z. and A. Garfinkel, *An advanced algorithm for solving partial differential equation in cardiac conduction.* IEEE Trans Biomed Eng, 1999. **46**(9): p. 1166-8.
13. Moreno, J.D., P.C. Yang, J.R. Bankston, E. Grandi, D.M. Bers, R.S. Kass, and C.E. Clancy, *Ranolazine for congenital and acquired late INa-linked arrhythmias: in silico pharmacological screening.* Circ Res, 2013. **113**(7): p. e50-61.
14. ten Tusscher, K.H. and A.V. Panfilov, *Alternans and spiral breakup in a human ventricular tissue model.* Am J Physiol Heart Circ Physiol, 2006. **291**(3): p. H1088-100.
15. Zhu, W., A. Mazzanti, T.L. Voelker, P. Hou, J.D. Moreno, P. Angsutararux, K.M. Naegle, S.G. Priori, and J.R. Silva, *Predicting Patient Response to the Antiarrhythmic Mexiletine Based on Genetic Variation.* Circ Res, 2019. **124**(4): p. 539-552.
16. Sheets, M.F. and D.A. Hanck, *Outward stabilization of the S4 segments in domains III and IV enhances lidocaine block of sodium channels.* J Physiol, 2007. **582**(Pt 1): p. 317-34.

17. Rudokas, M.W., Z. Varga, A.R. Schubert, A.B. Asaro, and J.R. Silva, *The Xenopus oocyte cut-open vaseline gap voltage-clamp technique with fluorometry.* J Vis Exp, 2014(85).
18. Varga, Z., W. Zhu, A.R. Schubert, J.L. Pardieck, A. Krumholz, E.J. Hsu, M.A. Zaydman, J. Cui, and J.R. Silva, *Direct Measurement of Cardiac Na+ Channel Conformations Reveals Molecular Pathologies of Inherited Mutations.* Circ Arrhythm Electrophysiol, 2015. **8**(5): p. 1228-39.
19. Zhu, W., Z. Varga, and J.R. Silva, *Molecular motions that shape the cardiac action potential: Insights from voltage clamp fluorometry.* Prog Biophys Mol Biol, 2016. **120**(1-3): p. 3-17.
20. Lu, H.R., J. Rohrbacher, E. Vlaminckx, K. Van Ammel, G.X. Yan, and D.J. Gallacher, *Predicting drug-induced slowing of conduction and pro-arrhythmia: identifying the 'bad' sodium current blockers.* Br J Pharmacol. **160**(1): p. 60-76.
21. Ruan, Y., N. Liu, and S.G. Priori, *Sodium channel mutations and arrhythmias*, in *Nat Rev Cardiol*. 2009. p. 337-48.
